# Cholesterol-driven pathological astrocytic responses in diabetes-associated cognitive impairment through astrocytic SCAP accumulation and NF-κB–C3 signaling modulation

**DOI:** 10.1038/s12276-025-01534-w

**Published:** 2025-09-29

**Authors:** Tong Niu, Shaohua Wang, Haoqiang Zhang, Wenwen Zhu, Kunyu Liu, Xueling Zhou, Ruoyu Sun, Diejing Niu, Yang Yuan

**Affiliations:** 1 Department of Endocrinology, Affiliated Zhongda Hospital of Southeast University, Nanjing, China; 2 School of Medicine, Southeast University, Nanjing, China; 3 Department of Endocrinology, The First Affiliated Hospital of USTC, Division of Life Sciences and Medicine, University of Science and Technology of China, Hefei, China; 4 HuZhou Central Hospital, Huzhou, China

**Keywords:** Astrocyte, Diabetes complications

## Abstract

The diabetic environment, characterized by hyperglycemia, advanced glycation end products and cerebral insulin resistance, triggers pathological astrocytic responses that contribute to cognitive decline in diabetes-associated cognitive impairment. Cholesterol accumulation in the brain, particularly in astrocytes, contributes to this pathological process. SCAP, a cholesterol sensor involved in lipid imbalances, regulates metabolic diseases, but its role in astrocytes remains unclear. C57BL/6J wild-type and astrocyte-specific SCAP knockout mice were fed a high-fat diet and treated with streptozotocin to induce type 2 diabetes mellitus (T2DM). Behavioral tests and hippocampal histology were performed at 28 weeks. We investigated the NF-κB–C3 signaling pathway to elucidate how SCAP induces pathological astrocytic responses under diabetic conditions. Cognitive function was assessed in patients with T2DM using the Montreal Cognitive Assessment (MoCA) and the mini-mental state examination (MMSE). We found elevated SCAP expression in the astrocytes of T2DM mice, correlated with cognitive dysfunction, impaired synaptic plasticity and altered astrocyte morphology. These effects were mitigated in astrocyte-specific SCAP knockout mice. SCAP elevation activates NF-κB by recruiting IκBα to the Golgi apparatus, promoting C3 transcription. Conversely, the inhibition of SCAP suppressed NF-κB activation. In patients with T2DM, serum C3 levels were higher in those with mild cognitive impairment, showing a U-shaped correlation with low-density lipoprotein-cholesterol (LDL-C) levels. These findings uncover a critical regulatory axis underlying astrocytic dysfunction, where SCAP mediates pathological astrocytic responses via the NF-κB–C3 pathway, with the Golgi acting as the platform for SCAP-driven activation. Here we highlight the interaction between cholesterol disorders and pathological astrocytic responses, presenting SCAP as a potential target for therapeutic intervention in diabetes-associated cognitive impairment.

**Figure:**
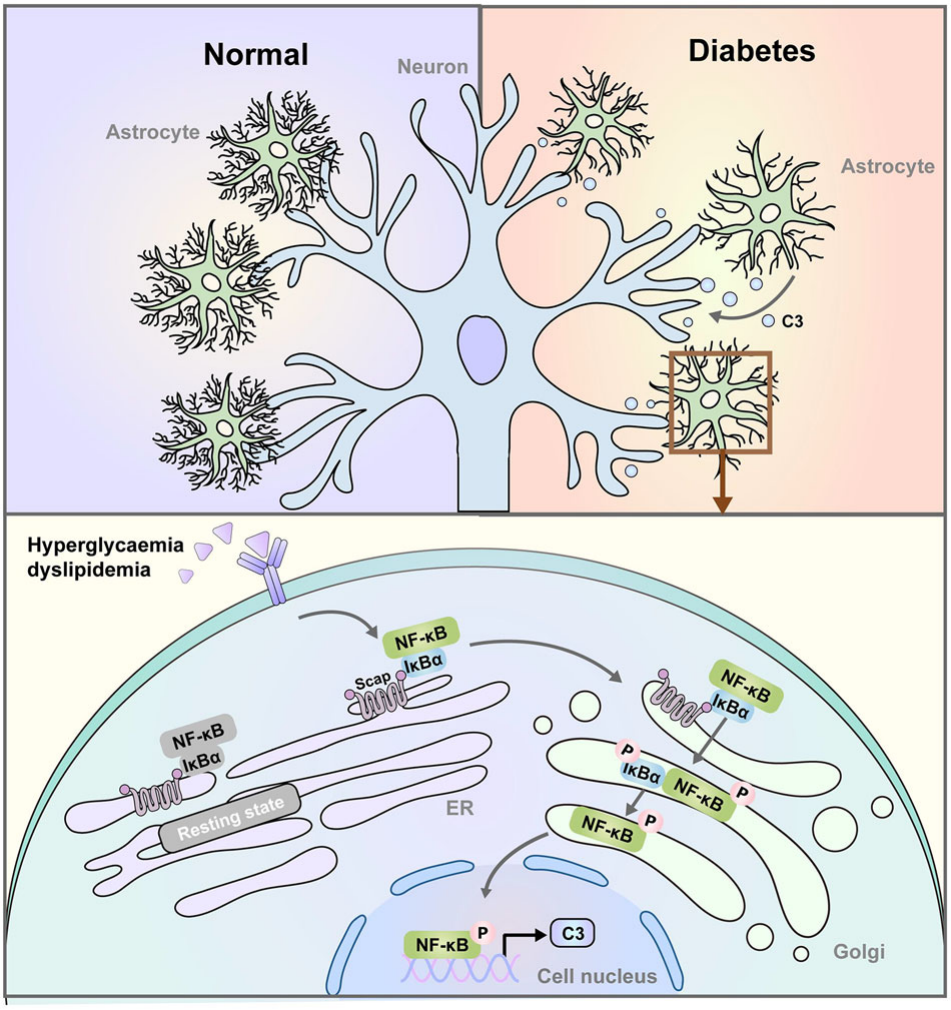
**Research Hypothesis Illustration: SCAP and complement C3 play a role in cholesterol-driven astrocyte responses in diabetes-associated cognitive impairment.** Astrocytic SCAP expression is abnormally increased in HFD/STZ-induced diabetic mice, impairing neuronal synaptic plasticity by activating the IκBα/NF-κB/C3 signalling pathway. Upregulated SCAP in astrocytes directly binds to IκBα, increasing its activation in the Golgi apparatus, which promotes NF-κB nuclear translocation and triggers complement C3 transcriptional activation and inflammatory immune responses, ultimately leading to neuronal and cognitive damage.

**Research Hypothesis Illustration: SCAP and complement C3 play a role in cholesterol-driven astrocyte responses in diabetes-associated cognitive impairment.** Astrocytic SCAP expression is abnormally increased in HFD/STZ-induced diabetic mice, impairing neuronal synaptic plasticity by activating the IκBα/NF-κB/C3 signalling pathway. Upregulated SCAP in astrocytes directly binds to IκBα, increasing its activation in the Golgi apparatus, which promotes NF-κB nuclear translocation and triggers complement C3 transcriptional activation and inflammatory immune responses, ultimately leading to neuronal and cognitive damage.

## SCAP regulates astrocytic dysfunction in diabetic cognitive decline

Diabetes is a growing global health issue, with over 640 million people expected to be affected by 2040. This study explores the link between diabetes, cholesterol metabolism and brain function. Researchers used diabetic mice to study how changes in cholesterol levels affect learning and memory. They focused on a protein called sterol regulatory element binding protein cleavage-activating protein (SCAP), which helps regulate cholesterol in cells. The article found that high cholesterol and glucose levels in diabetic mice led to brain changes that impaired learning and memory. The team discovered that SCAP plays a crucial role in these changes by affecting astrocytes. When SCAP was removed from astrocytes, the mice showed improved brain function and less cognitive decline. The findings suggest that targeting SCAP could be a new way to treat cognitive issues in diabetes.

This summary was initially drafted using artificial intelligence, then revised and fact-checked by the author.

## Introduction

The global prevalence of diabetes mellitus (DM) is projected to exceed 640 million by the year 2040^1,2^. One of the major neurodegenerative comorbidities of DM is diabetes-associated cognitive impairment (DACI), which affects ~13.5% of patients with diabetes, with prevalence rates exceeding 24.2% in individuals over the age of 75 years^3,4^. DACI is characterized by cognitive decline, which ranges from mild cognitive impairment (MCI) to dementia, the latter representing the most severe form and the second leading cause of death among patients with diabetes^5,6^. These cognitive impairments impede glycemic control, thereby increasing the risk of life-threatening complications such as hypoglycemia, hyperglycemic hyperosmotic coma and ketoacidosis. Therefore, understanding the complex pathophysiology underlying cognitive decline in individuals with diabetes represents an urgent research priority.

Patients with diabetes frequently exhibit alterations in cholesterol metabolism^7,8^. Previous studies have shown that insulin resistance and hyperglycemia can disrupt cholesterol metabolism in the brain^9^. Furthermore, chronic hypercholesterolemia, which begins in midlife and persists into older age, is associated with an increased risk of Alzheimer’s disease (AD)^10–12^, particularly as these hypercholesterolemia often overlap with obesity, metabolic syndrome and diabetes. Our previous studies demonstrated that patients with type 2 DM (T2DM) are more likely to exhibit abnormal lipid metabolism, especially cholesterol production, and metabolic disorders in the brain^13–15^. Collectively, these findings underscore an important relationship among diabetes, cholesterol metabolism and cognitive decline.

Sterol regulatory element binding protein (SREBP) cleavage-activating protein (SCAP) is a cholesterol sensor that regulates intracellular cholesterol homeostasis through signal transduction^16–18^. The dysregulation of SCAP impairs cholesterol homeostasis and lipid metabolism, contributing to metabolic diseases such as obesity, T2DM and cardiovascular disorders. Previous studies have demonstrated that SCAP is involved in the molecular mechanisms linking cholesterol metabolism directly to NLRP3 inflammasome activation^19^ and nuclear factor kappa B (NF-κB) signaling^20^. Inflammatory factors can further increase SCAP expression, promoting the translocation of the SCAP/SREBP2 complex from the endoplasmic reticulum (ER) to the Golgi apparatus, which disrupts cholesterol homeostasis and contributes to conditions such as atherosclerosis^21,22^ and nonalcoholic fatty liver disease^23^. SCAP dysfunction also triggers an inflammatory activation in macrophages^24,25^. These findings suggest that SCAP is a key intermediary linking cholesterol metabolism to inflammation, making it an attractive therapeutic target for metabolic diseases. Moreover, abnormal cholesterol metabolism is associated with the initiation of inflammation, amyloid-beta production and accumulation, tau protein phosphorylation and synaptic damage^26,27^. However, the impact of cholesterol metabolism on learning and memory in diabetic mice remains underexplored, and the role of SCAP in the pathophysiology of DACI is yet to be fully elucidated.

Astrocytes, essential for maintaining central nervous system (CNS) homeostasis and defense, play a vital role in cholesterol synthesis and metabolism within the brain^28,29^. Emerging evidence suggests that astrocytes are highly responsive to lifestyle changes and that their adaptability and plasticity may be crucial for favorable patient outcomes. Astrocyte morphology is particularly sensitive to environmental conditions^30,31^. For example, chronic overnutrition or sustained astrocytic IKKβ/NF-κB activation in astrocytes has been shown to induce astrocytic process shortening, potentially contributing to the development of metabolic syndrome^32^. Conversely, caloric restriction enhances astrocyte dendritic complexity, expands astrocyte territories and increases the volume of perisynaptic leaflets, thereby improving glutamate clearance and K^+^ buffering, ultimately supporting synaptic plasticity^33^. Given the high plasticity of astrocytes, they are likely intermediaries linking lifestyle factors, such as chronic overnutrition, to brain function. However, the role of astrocyte responses to glycolipid toxicity in DACI has been largely unexplored.

In neurodegenerative diseases, such as AD, astrocytes undergo complex changes, ranging from atrophy with functional loss to reactive astrogliosis characterized by hypertrophy, which leads to neurotoxicity and impairments in synaptic support^34,35^. These transformations exacerbate neurodegeneration and reduce the ability of astrocytes to maintain synaptic plasticity^36–38^. Astrocytes, which are highly plastic cells, continuously monitor the brain environment and are critical for maintaining biochemical homeostasis, remodeling neuronal circuits and orchestrating adaptive brain responses^39^. Although not primarily immune cells, astrocytes can detect neurotoxic danger-associated molecules early in disease progression and undergo changes to become disease-associated astrocytes^40^. The abundance of disease-associated astrocytes increases substantially as the disease progresses, contributing to the inflammation and neurotoxicity^41^. However, the specific role of diabetes-induced astrocyte dysfunction in driving neuronal damage in DACI remains largely unexplored.

Building on prior research on SCAP and its involvement in metabolic diseases, we aimed to investigate whether astrocytic SCAP is a crucial mediator between lipid metabolism and astrocyte dysfunction in DACI. This study evaluated the effects of abnormal glucose and lipid metabolism on learning and memory behaviors while exploring the underlying mechanisms. Specifically, we focused on cerebral cholesterol levels, hippocampal neuroplasticity, astrocyte morphology and SCAP expression in astrocytes. Our findings suggest that astrocytic SCAP may serve as a central mediator bridging cerebral cholesterol metabolic disturbances and astrocyte dysfunction in DACI.

## Materials and methods

### Mouse models

**Fig. 1:**
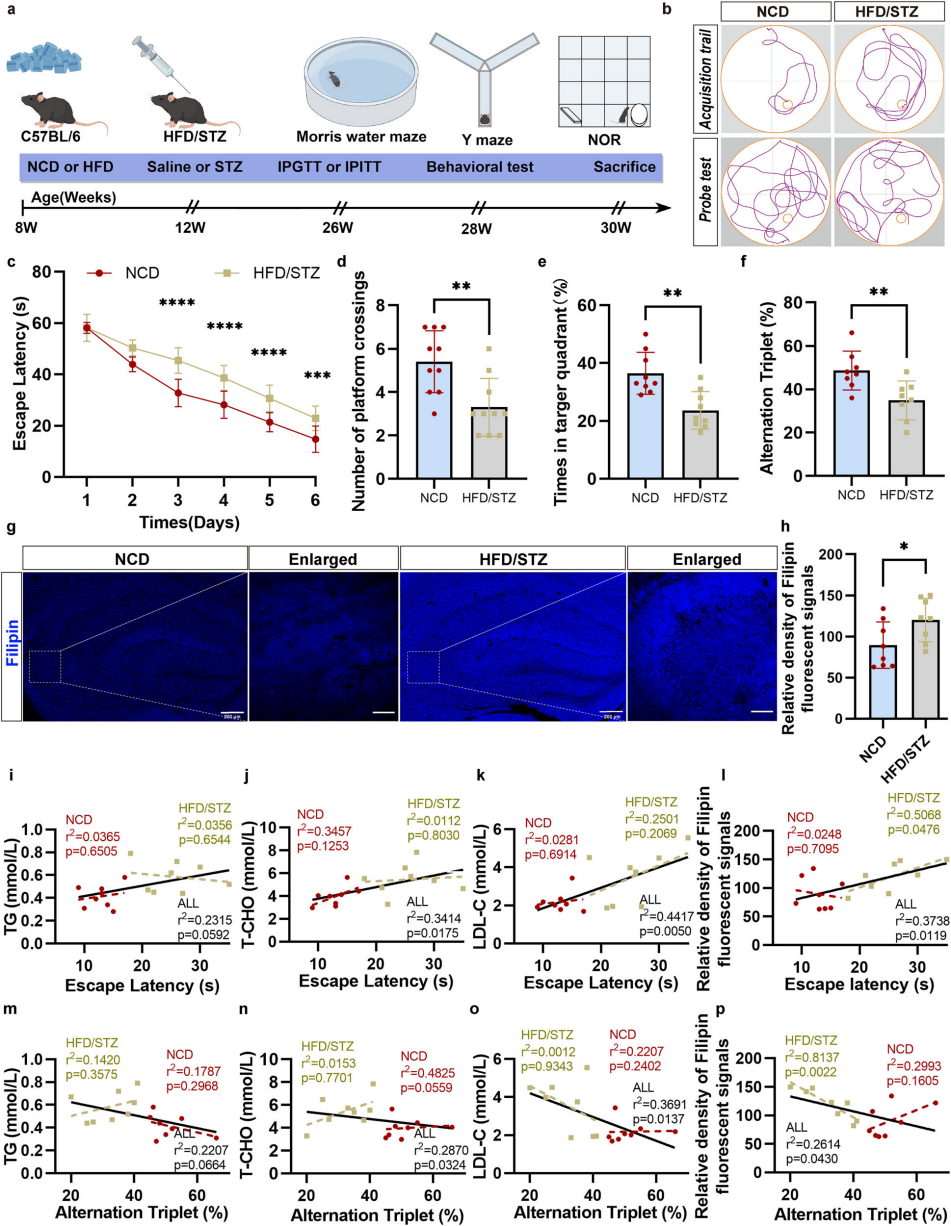
HFD consumption combined with STZ injection aggravates learning and memory impairments in mice. **a** A schematic illustration of the chronological order of HFD feeding, STZ injection, intraperitoneal glucose tolerance test (IPGTT) testing, intraperitoneal insulin tolerance test (IPITT) testing and cognitive testing. **b**, The MWM test traces for NCD and HFD/STZ mice (*n* = 8 per group). **c** The HFD/STZ mice showed delayed MWM escape latency compared with NCD mice (****P < 0.0001 days 3–5, ***P = 0.006 day 6). **d**, **e** The number of platform crossings (**d**) and target quadrant retention time (%) (**e**) during the probe trial of the MWM test were lower in the HFD/STZ group than in the NCD group (**P = 0.0032 in **d**, **P = 0.0012 in **e** between the NCD and HFD/STZ groups). **f** The lower alternated triplets (%) in the Y-maze for HFD/STZ mice (**P = 0.0084; *n* = 8 per group). **g**, **h** The brain sections from the NCD-fed or HFD/STZ-induced mice were stained with the filipin probe (**g**); the immunofluorescence intensity of filipin^+^ cholesterol was quantified and shown (**h**) (*n* = 6 per group). **i**–**l** Linear regressions of MWM escape latency against: i serum TG, j T-CHO (total cholesterol), k LDL-C, and l hippocampal free cholesterol (Filipin fluorescence). **m**–**p** Linear regressions of Y-maze alternation (%) against the same indices: m TG, n T-CHO, o LDL-C, and p hippocampal free cholesterol. NCD (red circles) and HFD/STZ (yellow squares) are shown separately. The data are means ± s.e.m. *P < 0.05, **P < 0.01, ***P < 0.001, ****P < 0.0001. The two-tailed unpaired Student’s *t*-test was used in **d**–**f** and **h** and one-way ANOVA with Tukey’s post hoc test in **c**, and correlations were assessed via linear regression in **i**–**p**. See also Supplementary Fig. 1 for more information.

All experimental procedures followed the Animal Research: Reporting of In Vivo Experiments guidelines, adhered to animal ethics standards and were approved by the Institutional Animal Care and Use Committee of Zhongda Hospital and Southeast University, China (approval no. 20210301022). Astrocyte-specific SCAP knockout (KO) mice (Aldh1l1-CreERT2 SCAP flox/flox mice) were generated by breeding Aldh1l1-CreERT2 mice (Cyagen Biosciences) with SCAP flox/flox mice (Jackson Laboratory). We refer to mice carrying the Aldh1l1-CreERT2 genes and SCAP flox/flox as ‘AS cKO’ and their littermates without the Aldh1l1-CreERT2 gene as ‘Cre-, f/f’. Littermates without the SCAP flox/flox are designated wild-type (WT) mice. The 7-week-old male C57BL/6J mice were obtained and used to establish a T2DM model through high-fat diet (HFD, 60% fat) feeding and low-dose streptozotocin (STZ) injections (40 mg/kg for 5 days). Mice fed a normal chow diet (NCD, 10% fat) served as controls. After a 1-week acclimation, 8-week-old mice were divided into HFD and NCD groups for 4 weeks. Following a 12-h fast, HFD-fed mice received STZ, while NCD mice received citric acid buffer^42^. The diabetic mice, identified by fasting blood glucose >11.1 mmol/l in three consecutive tests, continued on the HFD for 16 weeks^43^. Behavioral tests assessed cognitive performance in 28-week-old mice. The experimental process is shown in Fig. 1a.

### Cell culture

Primary neuron culture: neurons from postnatal day 0 (P0) mouse cortices were isolated using the Worthington Papain Dissociation System^44^. The cortices were digested with papain and DNase, triturated into single-cell suspensions and filtered. The cells were pelleted, resuspended in suspension media and layered over a density gradient for centrifugation. The neurons were plated on poly-ʟ-lysine-coated dishes in neurobasal medium with B27, ʟ-glutamine and penicillin–streptomycin. For MAP2-synaptophysin (SYP) imaging, cells were plated at 3.5 × 10⁵ cells/well in 24-well plates. For immunoblotting, cells were plated at 1 × 10⁶ cells per well in six-well plates.

Primary astrocyte culture: hippocampal tissues from P2–3 mouse pups were digested with trypsin, filtered and plated in poly-ʟ-lysine-coated flasks containing DMEM, 10% FBS and penicillin–streptomycin. After 7–9 days, purified by shaking, astrocytes were seeded into culture plates for experimental use. Astrocytes were cultured on (Poly-D-lysine) PDL-coated plates or coverslips. To simulate glycolipid abnormalities in T2DM, cells were treated with Dulbecco’s modified Eagle medium (DMEM) containing 25 mM glucose and 100 μg/ml low-density lipoprotein cholesterol (LDL-C) for 24 h. The controls included DMEM with 5.5 mM glucose, 25 mM glucose or LDL-C. SCAP inhibition was achieved with 4-hydroxytamoxifen (1 μM in DMEM), applied for 48 h and changed with medium after 5 days for coculture or cell lysis. Neurons were pretreated with 10 μM C3aRA for 1 h before the coculture with astrocytes. All in vitro experiments were conducted in triplicate.

### Clinical subjects and assessments

To investigate risk factors for DACI, 202 patients with T2DM (76 with MCI and 126 without) were recruited. The inclusion criteria included ≥6 years of education and ≥3 years of T2DM. Patients with infections, inflammation or other mental disorders were excluded. Cognitive function was assessed using the Montreal Cognitive Assessment (MoCA) and mini-mental state examination (MMSE). Correlation analyses were conducted between MoCA scores and serum metabolic indicators. The ethical approval was obtained (approval no. 2023ZDSYLL435-P01), and informed consent was provided by all participants. Serum C3 levels were measured using a Human C3 ELISA Kit (Jin Yibai) following the manufacturer’s instructions.

Detailed descriptions of the in vivo and in vitro analyses, mouse model protocols, preparation of reagents and samples, behavioral tests, cell isolation and magnetic-based cell sorting, enzyme-linked immunosorbent assay (ELISA), primary neuron and astrocyte extraction, culture and treatment conditions, neuron–astrocyte coculture, astrocyte-conditioned medium collection, western blotting, protein coimmunoprecipitation, Cleavage Under Targets & Release Using Nuclease (CUT&RUN) assay and analysis, immunofluorescence staining, astrocyte morphology analysis, synaptic imaging and quantification and Golgi staining^45^ and morphometric analyses are described in the Supplementary Materials and Methods. The reagents and antibodies used in this research including the corresponding dilutions, primers and software, along with their sources and research resource identifier numbers, are detailed in the Supplementary Data.

### Statistical analysis

Statistical significance between groups was determined using GraphPad Prism 9.0 (GraphPad) software. The data are presented as means ± standard errors of the mean (s.e.m.). The data were first evaluated for normality and equal variance, after which suitable statistical tests were selected on the basis of these assessments. The unpaired *t*-test was used to determine the significance of differences between two groups of normally distributed data, whereas the Mann–Whitney *U* test was used to analyze differences in nonnormally distributed data. One-way analysis of variance (ANOVA), followed by Tukey’s post hoc test, was used to assess the significance among three or four groups. In addition, a repeated measures two-way ANOVA was applied to compare data from different groups at various time points, allowing the assessment of changes in body weight, blood glucose and Morris water maze (MWM) spatial training over time. Curve fitting was performed to examine the relationship between LDL-C (or total cholesterol) and MoCA. Pearson or partial correlation analyses were performed to evaluate the association between serum C3 levels and neuropsychological tests, as well as serum lipids, with adjustments for age, gender and education duration. Binary logistic regression was used to identify independent risk factors for MCI. All significant statistical results are indicated within the figures with the following conventions: *P < 0.05, **P < 0.01, ***P < 0.001, ****P < 0.0001. All experiments were performed at least three times, and the findings were replicated in mice and cell cultures in each experiment.

## Results

### Cognitive dysfunction in HFD/STZ-induced diabetic mice is associated with cholesterol deposition in the hippocampus

All mice were weighed at the start and every 2 weeks thereafter. Initially, fasting body weight was comparable across groups. After 4 months of dietary and glycemic manipulation, the HFD/STZ-induced diabetic mice exhibited significantly increased body weights compared with the controls, reflecting an obesogenic effects of HFD/STZ (Supplementary Fig. 1a). HFD/STZ mice also exhibited elevated fasting glucose (Supplementary Fig. 1b), impaired glucose tolerance, insulin resistance (Supplementary Fig. 1c,d) and dyslipidemia with increased serum triglycerides (TG), total cholesterol and LDL-C levels (Supplementary Fig. 1e–h), indicating metabolic disturbances.

The timeline of diabetes modeling, glucose tolerance and behavioral tests is shown in Fig. 1a. Behavioral tests, including MWM, Y-maze and novel object recognition (NOR), demonstrated that the HFD/STZ group exhibited prolonged escape latency (Fig. 1b, c), fewer platform crossings (Fig. 1d) and reduced time in the target quadrant (Fig. 1e) in the MWM, indicating impaired spatial learning and memory, with no differences in swimming velocity (Supplementary Fig. 1i). The Y-maze test showed reduced spontaneous alternation (%) in HFD/STZ mice (Fig. 1f), with no significant changes in total arm entries or distance traveled (Supplementary Fig. 1j, k). In the NOR test, HFD/STZ mice had a lower recognition index (Supplementary Fig. 1l, m), confirming the cognitive deficits.

Filipin staining revealed increased hippocampal free cholesterol (FC) levels in HFD/STZ mice compared with the NCD group (Fig. 1g, h). The linear regression analysis showed that although serum total cholesterol, LDL-C and hippocampal FC levels correlated with learning and memory deficits across all groups (Fig. 1i–p), hippocampal FC deposits were the primary factor linked to these deficits in HFD/STZ mice, independent of serum lipid levels. These findings suggest hippocampal FC deposition predominates in DACI once obesity or hyperglycemia reaches a threshold.

### HFD/STZ-induced mice exhibit impaired neuronal synaptic plasticity and a neurotoxic astrocyte phenotype

**Fig. 2:**
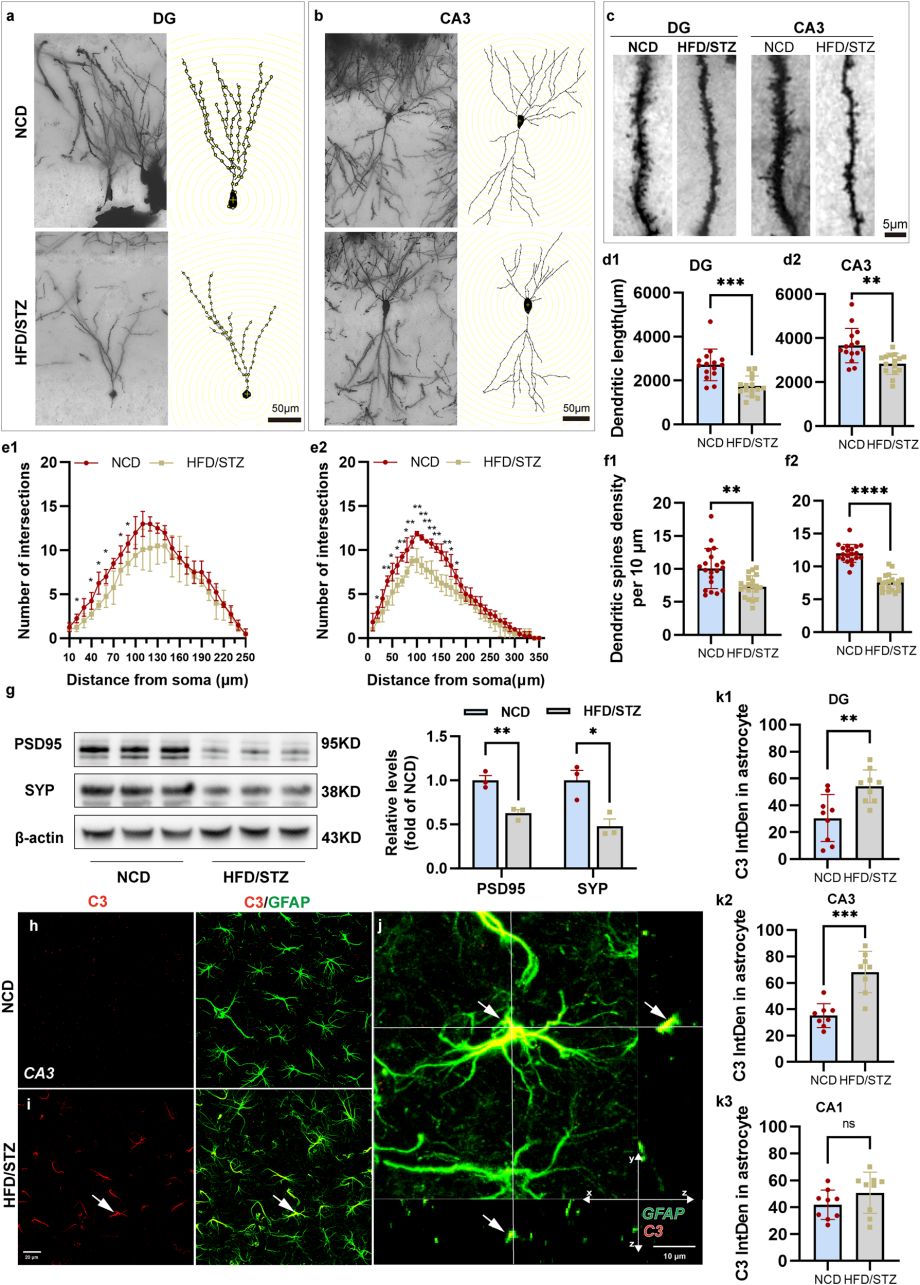
HFD/STZ induced neuronal morphological changes and a neurotoxic astrocyte phenotype in the hippocampus. **a**, **b** The representative traces of granule cells in the DG (**a**) and pyramidal cells in the CA3 region (**b**) along with a graphic description of Sholl analysis parameters. Scale bars, 50 µm. **c** The representative photomicrographs of the secondary apical branches of granule neurons in the DG neurons in the CA3 region. **d**, **e** The Sholl analysis showed reduced total dendritic length in the DG (d1) and CA3 (d2), as well as reduced dendritic complexity in the DG (e1) and CA3 (e2), in HFD/STZ mice compared with NCD controls. **f** The number of dendritic spines per 10 µm in the DG (f1) and CA3 (f2) regions was reduced in HFD/STZ-induced mice (*n* = 20 per group). **g** The representative immunoblots of the synaptic proteins PSD95 and SYP (*n* = 3 per group). **h**, **i** The representative maximum intensity projections of confocal images of GFAP- and C3-labeled astrocytes from the NCD (**h**) and HFD/STZ (**i**) groups. Scale bars, 20 µm. **j** The confocal orthogonal view of C3 encapsulated by astrocytes in **i**. C3, indicated by the arrow in the *x*–*y* projection, can be visualized within astroglia in the orthogonal *x*–*z* and *y*–*z* projections (also indicated by the arrow). Scale bars, 10 µm. **k** The average integrated fluorescence intensity (IntDen) of C3 colocalized with GFAP per astroglia in the DG (k1), CA3 (k2) and CA1 (k3). All of the data are presented as the mean ± s.e.m. Two-tailed unpaired Student’s *t*-tests were used. *P < 0.05, **P < 0.01, ***P < 0.001, ****P < 0.0001. n.s., not significant. See also Supplementary Fig. 2 for further information.

Alterations in hippocampal neuron architecture are known to contribute significantly to cognitive disorders. We analyzed the dendritic complexity (length and arborization) and spine density of dentate gyrus (DG) granule cells, CA3 and CA1 pyramidal cells using Golgi staining. A Sholl analysis revealed reduced dendritic complexity in DG (Fig. 2a) and CA3 (Fig. 2b) neurons in the HFD/STZ group, evidenced by decreased dendritic length (Fig. 2d) and intersections (Fig. 2e) with Sholl spheres. No significant changes were found in CA1 neurons (Supplementary Fig. 2a, b). The spine density was significantly reduced in the DG and CA3 regions of HFD/STZ mice (Fig. 2c, f) but remained unchanged in CA1 (Supplementary Fig. 2c). The synaptic protein analysis revealed significantly decreased PSD-95 and SYP levels in the HFD/STZ group compared with the NCD group (Fig. 2g). These results indicate that DACI mice have altered neuronal morphology and impaired synaptic plasticity.

**Fig. 3:**
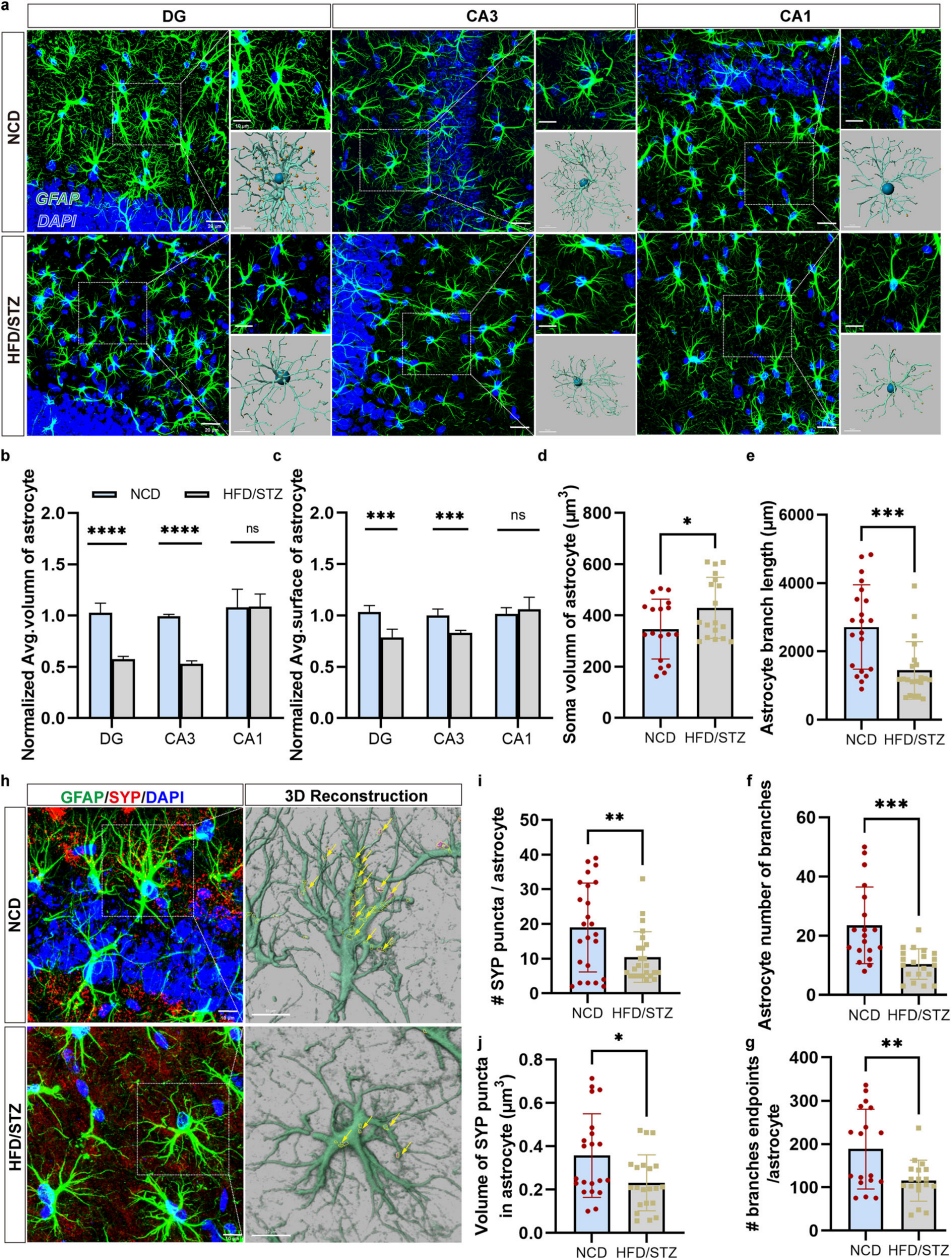
Hippocampal astrocytes in HFD/STZ-induced mice exhibited an atrophic morphology and decreased synaptic coverage. **a** Representative *z*-projection images of GFAP- and DAPI-labeled hippocampal subregions surveyed in all groups. Scale bar, 20 μm. The insets show higher magnifications (top) and 3D rendering (bottom) of the boxed areas in the respective panels. Scale bar, 10 μm. The brain tissues from five independent experiments were analyzed (*n* = 5 per group). **b**, **c** The quantification of astrocyte volume (**b**) and surface area (**c**) in hippocampal subregions. *n* = 4–5 cells/animal. **d**–**g** The quantification of the astrocyte soma volume (**d**), branch length (**e**), branch number (**f**) and branch endpoints (**g**). **h** The representative images of SYP coverage with astrocytes in the hippocampus in each group. Scale bar, 10 μm. The insets show the 3D rendering of boxed areas in the respective panels. **i**, **j** The quantification of the number (**i**) and volume (**j**) of SYP-positive puncta colocalized with GFAP per astrocyte in the hippocampus. The data are presented as the means ± s.e.m. A two-tailed unpaired Student’s *t*-test was used. n.s., nonsignificant. *P < 0.05, **P < 0.01, ***P < 0.001, ****P < 0.0001.

Astrocytes in neurodegenerative models often express neurotoxic ‘A1’ phenotype markers, including complement C3. Significantly elevated levels of C3 were detected in the hippocampus of HFD/STZ mice using western blotting and ELISA (Supplementary Fig. 2d, e). Immunofluorescence staining confirmed that C3 expression was notably increased in GFAP-positive astrocytes in the CA3 (Fig. 2h–j) and DG regions (left panel of Supplementary Fig. 2f), but not in CA1 (right panel of Supplementary Fig. 2f), suggesting a neurotoxic astrocyte phenotype in the DACI model. Further analysis showed that the mean integrated fluorescence intensity of C3, which colocalized with GFAP, was increased in the DG (Fig. 2k, (1)) and CA3 (Fig. 2k, (2)) regions of the hippocampus, whereas there was no significant change in the CA1 region (Fig. 2k, (3)). Furthermore, the colocalization analysis of GFAP and C3 within astrocytes, reflecting the proportion of C3 signals overlapping with GFAP-positive astrocytes, demonstrated a significantly increased colocalization in the HFD/STZ mice compared with controls (Supplementary Fig. 2g). Astrocyte density and activation were assessed via GFAP staining (Supplementary Fig. 2h). GFAP fluorescence intensity was significantly elevated across all hippocampal subregions of HFD/STZ mice (Supplementary Fig. 2i), whereas astrocyte density remained unchanged across subregions (Supplementary Fig. 2j), suggesting an enhanced astrocyte reactivity in the DACI model.

### Astrocyte responses to high-glucose and cholesterol treatment lead to impaired synaptic density and altered dendritic morphology in neurons in vitro

Hyperglycemia is known to trigger structural and functional brain abnormalities. To examine the impact of astrocytes under high-glucose and/or cholesterol conditions on neurons, an astrocyte–neuron coculture system was used (Supplementary Fig. 3a). Astrocytes were treated with DMEM containing either 5.5 or 25 mM glucose, in the presence or absence of 100 μg/ml LDL-C, for 24 h before coculture with neurons. Cocultured neuron immunostaining for SYP and MAP2 (Supplementary Fig. 3b) revealed a significant reduction in dendritic complexity (Supplementary Fig. 3c), total dendritic length (Supplementary Fig. 3d) and synaptic density (Supplementary Fig. 3e) in neurons cocultured with astrocytes treated with 25 mM glucose and LDL-C. Neither treatment alone affected synaptic density or dendritic morphology, suggesting that the combination of high glucose and elevated cholesterol mediates astrocyte-associated neuronal damage. The primary astrocytes treated with high-glucose and LDL-C showed an increased C3 expression (Supplementary Fig. 3f,g) and increased C3 secretion into the medium (Supplementary Fig. 3h), with immunofluorescence also confirming elevated astrocytic C3 levels (Supplementary Fig. 3i, j). High glucose alone did not induce these changes. These results suggest that hyperglycemia combined with cholesterol drives pathological astrocytic responses characterized by increased C3 expression and secretion in vitro.

To clarify whether the ‘pathological astrocytic response’ is ‘C3–GFAP-double-positive astrocytes’, we used astrocyte-neuron coculture to quantify neuronal damage associated with these cells, verifying their neurotoxicity. Our results (Supplementary Fig. 3k, l) showed that compared with neurons cocultured with control astrocytes (GFAP^+^/C3^−^), neurons cocultured with high-glucose and LDL-C-treated astrocytes (GFAP^+^/C3^+^) showed significantly elevated levels of the apoptotic proteins Bax and cleaved caspase-3 and significantly reduced levels of the antiapoptotic protein Bcl-2. These data suggest that pathological astrocytic responses expressing C3 are likely to cause neuronal apoptosis in vitro, supporting the neurotoxic nature of C3–GFAP-double-positive astrocytes.

### Astrocytes in the hippocampus of HFD/STZ-induced mice exhibited reduced complexity and synaptic coverage

Astrocytic morphology, which is closely associated with astrocytic function, was analyzed across hippocampal subregions using GFAP staining (Fig. 3a). Confocal imaging and quantitative analysis using Imaris revealed significantly atrophic astrocyte morphology in the DG and CA3 regions of HFD/STZ-induced diabetic mice compared with the NCD group. Astrocytes in HFD/STZ mice exhibited smaller volumes (Fig. 3b) and areas (Fig. 3c), larger soma volumes (Fig. 3d) and reduced branch length (Fig. 3e), branch number (Fig. 3f) and branch endpoints (Fig. 3g). These findings indicate pathological astrocytic responses induced by HFD intake and STZ injection consistent with aging-related atrophy.

The astrocytic atrophy reduces the synaptic coverage and support, possibly contributing to cognitive decline. The high-resolution imaging of synaptic markers showed significantly decreased volume and number of presynaptic puncta (SYP) associated with GFAP-positive astrocytes in HFD/STZ mice compared with NCD-fed mice (Fig. 3h–j), which positively correlated with reduced levels of hippocampal synaptic protein (Fig. 2g). Furthermore, the colocalization analysis of GFAP and SYP within astrocytes, reflecting the proportion of SYP signals overlapping with GFAP-positive astrocytes, demonstrated a significantly reduced colocalization in the HFD/STZ mice compared with controls (Supplementary Fig. 2k). These data demonstrate that astrocytes exhibit morphological atrophy and reduced presynaptic coverage under diabetic conditions, which may be related to the impairment of synaptic plasticity.

### Upregulation of SCAP in astrocytes is involved in astrocytic responses to pathological conditions in vivo and in vitro

Aberrant cholesterol accumulation and complement C3-driven inflammation are key events in the pathogenesis of DACI. Linear regression analysis showed that hippocampal FC deposition was negatively correlated with astrocyte volume in DM mice (Supplementary Fig. 4a). Given the close relationship between astrocytic morphology and function, these results suggest that a critical role for astrocytes in abnormal cholesterol metabolism and related neurological dysfunction is observed in DACI. In addition, SCAP, as a signaling hub for cholesterol metabolism and inflammation, is predominantly expressed in astrocytes and neurons. Based on previous observations of pathological astrocytic responses and abnormal cholesterol metabolism in the hippocampus in the hippocampus of DACI mice, we further explored astrocytic SCAP alterations and evaluated their potential contribution to DACI pathogenesis.

**Fig. 4:**
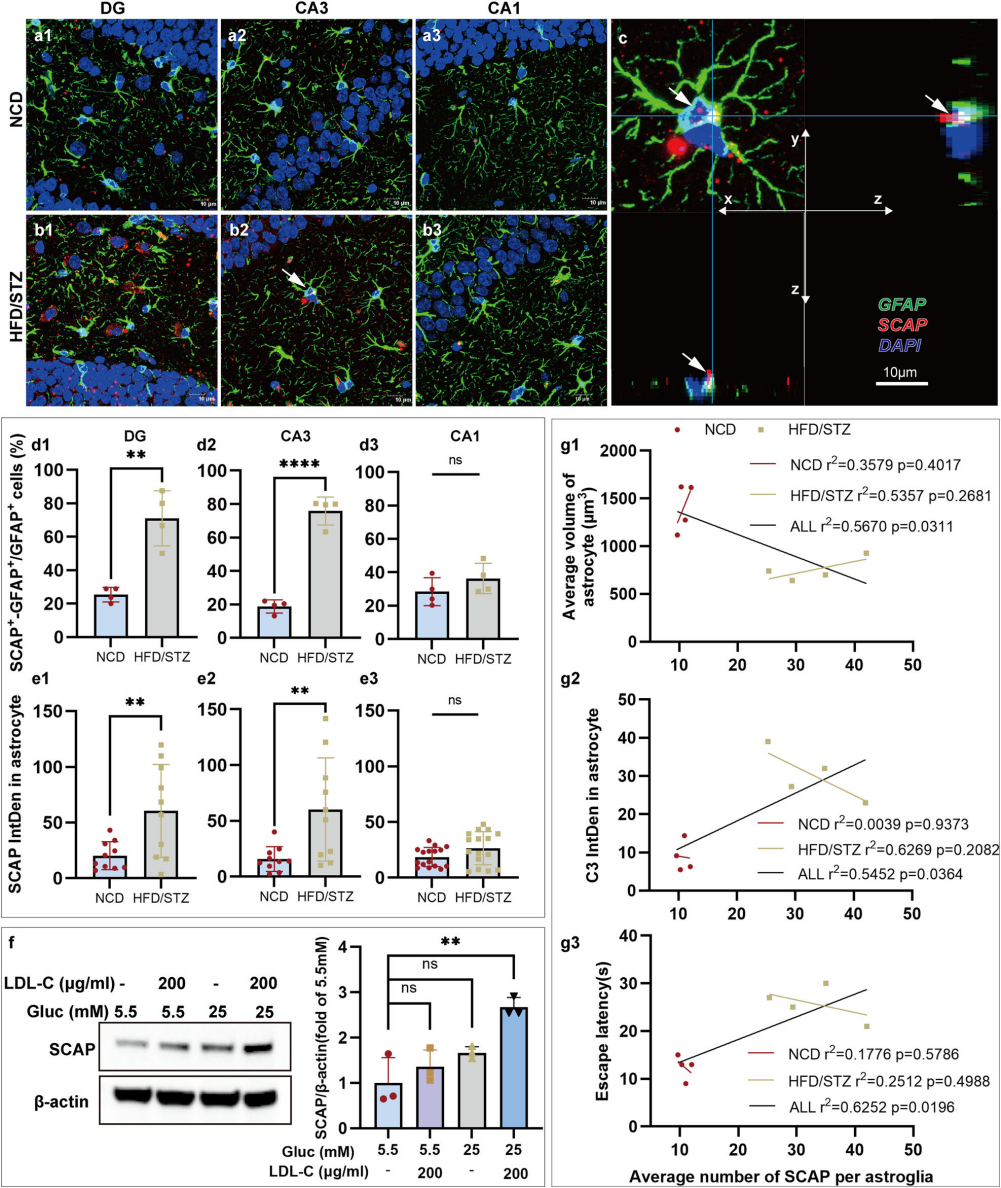
Astrocytes in the hippocampus of HFD/STZ-induced mice presented increased SCAP-stained puncta. **a**, **b** The representative confocal images of GFAP- and SCAP-stained DG (1), CA3 (2) and CA1(3) astrocytes in NCD (**a**) and HFD/STZ (**b**). Scale bars, 10 µm. **c** A confocal orthogonal view of the SCAP^+^ puncta encapsulated by the astrocyte in **b**, (2). The puncta indicated by arrows in the *x*–*y* projection can be visualized within the astrocyte in the orthogonal *x*–*z* and *y*–*z* projections (also indicated by arrows). Scale bars, 10 µm. **d** The average number of SCAP^+^ puncta colocalized with GFAP per astrocyte in the hippocampal subregions of interest ((1) DG, **P = 0.0017; (2) CA3, ****P < 0.0001; (3) n.s., CA1, between the NCD and HFD/STZ groups) (*n* = 4 per group). **e** The average integrated fluorescence intensity (IntDen) of SCAP colocalized with GFAP per astrocyte in the hippocampal subregions of interest ((1) DG, **P = 0.0092; (2) CA3, **P = 0.0085; (3) n.s., CA1, between the NCD and HFD/STZ groups) (*n* = 10 cells per group). **f** The SCAP expression in astrocytes with glucose (Gluc) (5.5 mM or 25 mM) or LDL-C treatment via western blot (*n* = 3 per experiment). **g** The linear regression of SCAP^+^ astrocytes versus (1) astrocyte volume, (2) C3 intensity and (3) escape latency (red, NCD; yellow, HFD/STZ; black, both). Data are mean ± s.e.m. One-way ANOVA with Tukey’s multiple comparisons test was performed for **f**. The Student’s *t*-test (two-sided) was performed for **d** (1–3) and **e** (1–3). Correlations (**g** (1–3)) were assessed via linear regression. n.s., nonsignificant. *P < 0.05, **P < 0.01, ***P < 0.001, ****P < 0.0001.

To assess the astrocytic SCAP’s role in the cognitive impairment of HFD/STZ-induced mice, we analyzed *z*-stack images of GFAP- and SCAP-labeled astrocytes in hippocampal subregions of NCD and HFD/STZ mice (Fig. 4a, b). SCAP-positive puncta in astrocytes were confirmed via three-dimensional (3D) projections (Fig. 4c). HFD/STZ-induced mice showed greater SCAP puncta numbers and fluorescence intensity within astrocytes in DG (**P = 0.0017 and **P = 0.0092) (Fig. 4d, e, (1)) and CA3 (****P < 0.0001 and **P = 0.0085) (Fig. 4d, e, (2)) regions than NCD-fed mice, with an increasing trend but no significant changes in CA1 (Fig. 4d, e, (3)). Concurrently, the colocalization analysis of GFAP and SCAP within astrocytes, reflecting the proportion of SCAP-positive signals overlapping with GFAP, revealed significantly increased colocalization in the DG (*P* = 0.007, Supplementary Fig. 4b, (1)) and CA3 (*P* = 0.0087, Supplementary Fig. 4b, (2)) regions of HFD/STZ mice compared with the NCD group, whereas there was no significant difference in the CA1 region (Supplementary Fig. 4b, (3)). The high-glucose and cholesterol treatment also significantly elevated SCAP levels in primary astrocytes (Fig. 4f), suggesting that metabolic disturbances associated with hyperglycemia and cholesterol exposure may drive astrocytic SCAP accumulation. The linear regression showed significant correlations between SCAP^+^ astrocytes and pathological astrocytic responses (decreased astrocyte volume: *P = 0.0311 (Fig. 4g, (1)) and increased fluorescence intensity of C3: *P = 0.0364 (Fig. 4g, (2))) as well as neurobehavioural indicators (prolonged escape latency in MWM (*P = 0.0196) (Fig. 4g, (3))) when including all mice. These findings highlight a significant association of SCAP with pathological astrocytic responses and behavioral impairments, suggesting that SCAP-positive astrocytes may contribute causally to the pathogenesis of DACI in HFD/STZ-induced diabetic mice.

### Astrocytic SCAP deletion abrogates HFD/STZ-mediated changes in hippocampal neuron morphology and reduced complexity

**Fig. 5:**
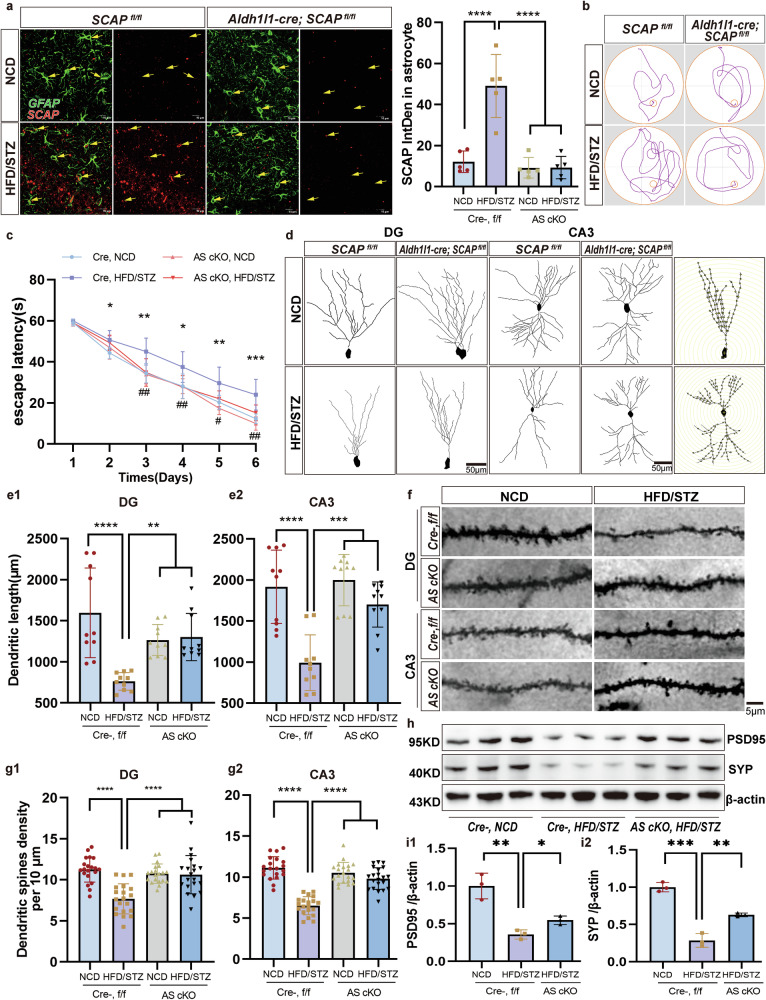
Astrocyte-specific SCAP ablation prevents HFD/STZ-induced alterations in neuronal morphology and synapse loss. **a** The representative confocal *z*-stack images of SCAP (red, yellow arrows) in astrocytes (GFAP, green) in the hippocampus in the four groups (Cre- NCD, Cre- HFD/STZ, AS cKO NCD and AS cKO HFD/STZ). The bar graphs show SCAP activation in astrocytes (*n* = 5 per group). Scale bars, 10 μm. **b** The representative samples of the path traveled during the probe test in the MWM test. **c** The escape latency in the MWM test. **d** The representative images of neuronal dendritic morphology in the four groups in **a**. Scale bar, 50 μm. **e** The quantification of total dendritic length in the (1) DG and (2) CA3 regions revealed a decrease in the Cre-HFD/STZ group compared with the Cre-NCD group ((1) ****P < 0.0001, (2) ****P < 0.0001 between Cre-NCD and Cre-HFD/STZ), which was increased after SCAP deletion ((1) **P = 0.004, (2) ***P = 0.0003 between Cre-HFD/STZ and AS-cKO-HFD/STZ). **f** Representative photomicrographs of granule neurons in the DG and pyramidal neurons in the CA3 region. **g** The number of dendritic spines per 10 µm of neurons in the (1) DG and (2) CA3 regions. **h**, **i** The representative immunoblots (**h**) and the quantification (**i**) of the following synaptic proteins: PSD95 and SYP (*n* = 3 per group) ((1)**P = 0.0035, (2) ***P = 0.0004 between Cre- NCD and Cre- HFD/STZ) ((1) *P = 0.0173, (2) **P = 0.0033 between Cre- HFD/STZ and AS cKO HFD/STZ). The data are expressed as the means ± s.e.m., and one-way ANOVA with Tukey’s multiple comparisons test was performed for all the statistical analyses. *P < 0.05, **P < 0.01, ***P < 0.001, ****P < 0.0001. No other statistical comparisons are significant unless indicated.

To test the hypothesis that astrocytes with upregulated SCAP expression mediate neuroplasticity deficits in HFD/STZ-induced DACI, astrocyte-specific inducible SCAP KO (AS cKO) mice were generated by crossing Aldh1l1-creERT2 mice with loxP-floxed SCAP mice (Supplementary Fig. 4d). Immunohistochemistry and reverse-transcription polymerase chain reaction confirmed specific SCAP inhibition in astrocytes (Fig. 5a), with no changes in SCAP expression in neurons (Supplementary Fig. 4e) or other tissues (Supplementary Fig. 4f). Astrocyte-specific SCAP KO, diabetes modeling and behavioral testing were performed in four groups mice: Cre-, f/f NCD; Cre-, f/f HFD/STZ; AS cKO NCD; and AS cKO HFD/STZ (Supplementary Fig. 4g). The colocalization analysis of GFAP and SCAP in astrocytes also showed that the colocalization degree of SCAP and GFAP in astrocytes in the AS cKO HFD/STZ group was significantly reduced compared with Cre-, HFD/STZ (Supplementary Fig. 4c). No differences in body weight (Supplementary Fig. 5a), fasting blood glucose (Supplementary Fig. 5b), glucose tolerance (Supplementary Fig. 5c) and lipid metabolism were detected between Cre-, HFD/STZ and AS cKO HFD/STZ groups (Supplementary Fig. 5d–g).

Learning and memory were evaluated using the MWM, Y-maze and NOR tests across four groups. In the MWM test, HFD/STZ-induced mice (Cre-, f/f) showed increased escape latency (Fig. 5b, c), reduced platform crossings (Supplementary Fig. 5j) and lower target quadrant retention time (Supplementary Fig. 5k) compared with NCD mice, but these deficits were mitigated in AS cKO HFD/STZ mice. Similarly, the alternation triplets in Y-maze (Supplementary Fig. 5m) and the recognition index in NOR test (Supplementary Fig. 5n, o) were impaired in Cre-, f/f HFD/STZ mice, but these deficits were improved in AS cKO HFD/STZ mice. There was no difference in the swimming speed and total arms entries among the four groups (Supplementary Fig. 5i, l).

Golgi staining revealed significantly reduced dendritic length (Fig. 5d) in the DG (Fig. 5e, (1)) and CA3 (Fig. 5e, (2)) regions, along with decreased dendritic spine density (Fig. 5f) in the DG (Fig. 5g, (1)) and CA3 (Fig. 5g, (2)) of Cre-, f/f HFD/STZ mice. These deficits were attenuated in AS cKO HFD/STZ mice. A Sholl analysis of pyramidal neurons showed decreased intersections with Sholl spheres in Cre-, f/f HFD/STZ mice, which were alleviated in AS cKO HFD/STZ mice (Supplementary Fig. 5h). Levels of synaptic proteins (PSD-95 and SYP) were significantly decreased in Cre-, f/f HFD/STZ mice but were preserved in AS cKO HFD/STZ mice (Fig. 5h, i). These findings demonstrate that astrocyte-specific SCAP elimination mitigates neuroplasticity deficits and learning and memory impairments caused by HFD/STZ-induced DACI.

### Astrocytic SCAP elimination alleviates HFD/STZ-mediated neurotoxicity and morphological damage to astrocytes

**Fig. 6:**
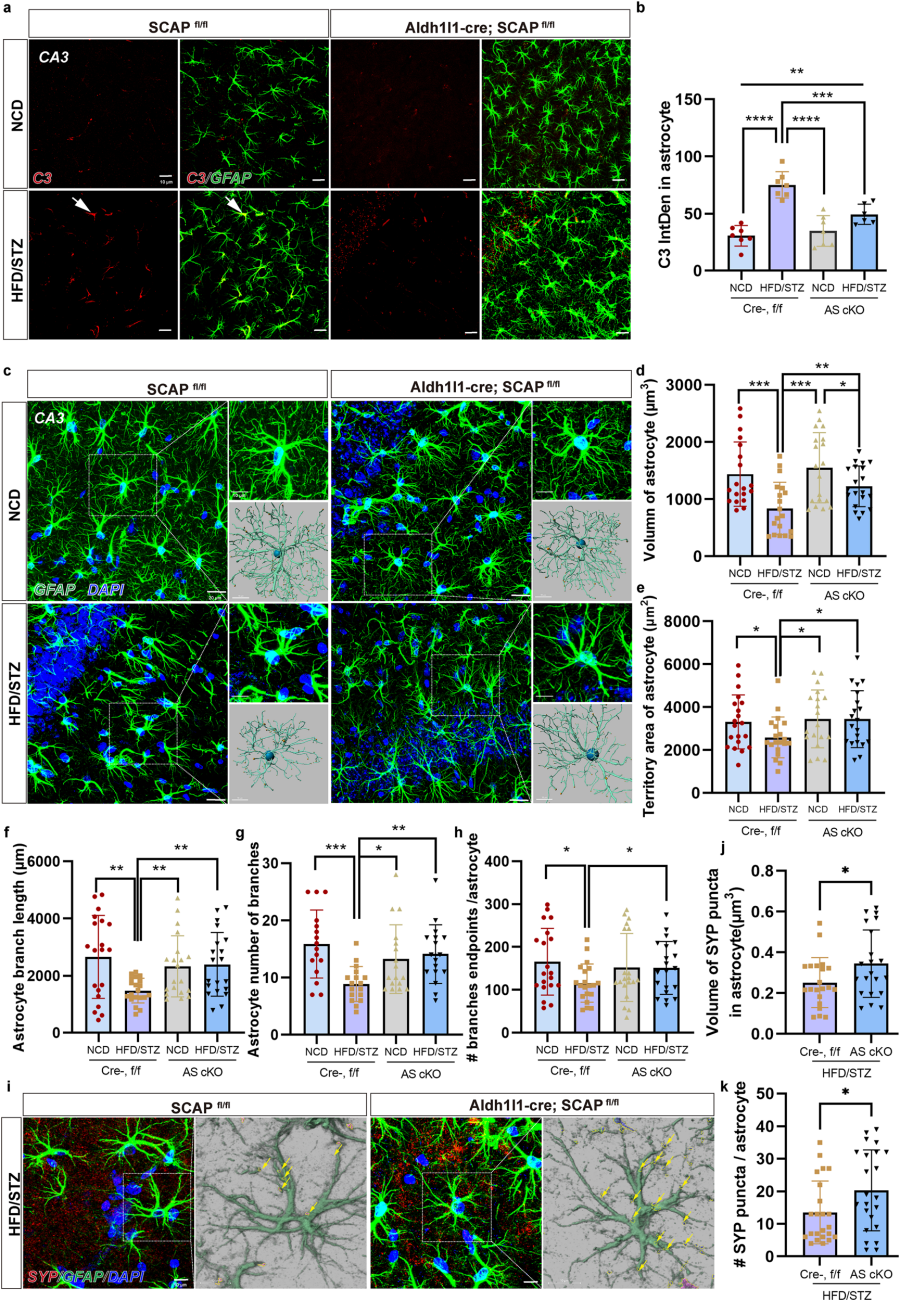
Astrocyte-specific SCAP ablation triggers a phenotypic switch in reactive astrocytes and attenuates astrocyte atrophy. **a** The representative maximum intensity projections of confocal images of GFAP- and C3-labeled astrocytes from Cre- NCD, Cre- HFD/STZ, AS cKO NCD and AS cKO HFD/STZ. Scale bars, 10 µm. **b** The average integrated fluorescence intensity (IntDen) of C3 colocalized with GFAP per astrocyte in the hippocampus. **c** The representative *z*-projection images of GFAP- and DAPI-labeled hippocampal subregions surveyed in the four groups. Scale bar, 20 μm. Insets show higher magnifications (top) and 3D rendering (bottom) of the boxed areas in the respective panels. Scale bars, 10 µm. *n* = 5 per group. **d**, **e** The quantification of astrocyte volume (**d**) and surface area (**e**) in the hippocampal subregions; *n* = 5 per group, *n* = 4–5 cells per animal. **f**–**h** The quantification of astrocyte branch length (**f**), branch number (**g**) and branch endpoints (**h**); *n* = 5 per group, *n* = 4–5 cells per animal. **i** The representative images of SYP coverage with astrocytes in the hippocampus from each group. Scale bar, 10 μm. Insets show 3D rendering of boxed areas in the respective panels. **j**, **k** The quantification of SYP-positive puncta (**j**) and the number (**k**) of SYP-positive puncta colocalized with GFAP^+^ astrocytes in the hippocampus. The data are expressed as the means ± s.e.m. The one-way ANOVA with Tukey’s multiple comparisons test was performed for all the statistical analyses except **j** and **k**, where Student’s *t*-test (two-tailed) was performed. *P < 0.05, **P < 0.01, ***P < 0.001, ****P < 0.0001. No other statistical comparisons are significant unless otherwise indicated.

Building upon our previous findings that astrocytic SCAP content correlates with pathological astrocyte responses in HFD/STZ-induced mice, we further examined astrocytic neurotoxicity and morphological changes following astrocyte-specific SCAP deletion. Elevated C3 and C3AR protein levels observed in Cre-, f/f HFD/STZ mice were significantly attenuated in AS cKO mice (Supplementary Fig. 6a, b). A double immunofluorescence staining for GFAP and C3 revealed that the HFD/STZ-induced increase of astrocytic C3 expression in the CA3 (Fig. 6a, b) and DG regions (Supplementary Fig. 6c, d) observed in Cre-, f/f mice was effectively prevented in AS cKO mice, indicating SCAP’s involvement in astrocyte activation. At the same time, a colocalization analysis of GFAP and C3 within astrocytes (indicates the proportion of C3 signals colocalized with GFAP) also showed that the degree of colocalization was significantly reduced in AS cKO mice compared with Cre-, f/f HFD/STZ mice (Supplementary Fig. 6e).

The quantitative analysis of astrocyte morphology using Imaris software showed that HFD/STZ-induced astrocytes exhibited smaller volumes and areas in the CA3 (Fig. 6c–e) and DG regions (Supplementary Fig. 6f–h), which were ameliorated in AS cKO mice. Moreover, astrocytic complexity, as indicated by branch length and number, was reduced in Cre-, f/f HFD/STZ mice, but these deficits were restored by the astrocyte-specific SCAP deletion (Fig. 6f–h).

To assess the synaptic function, immunostaining for SYP and GFAP revealed increased SYP-positive puncta volume and number within SCAP-deficient astrocytes in HFD/STZ-induced mice (Fig. 6i–k). Furthermore, colocalization analysis of GFAP and SYP within astrocytes, indicating the proportion of SYP-positive signals overlapping with GFAP, also showed significantly increased colocalization in AS cKO mice compared with Cre-, f/f HFD/STZ mice (Supplementary Fig. 6i). This is also consistent with the increased levels of hippocampal synaptic proteins in AS cKO mice (Fig. 5h). These findings demonstrate that SCAP elimination in astrocytes mitigates astrocytic neurotoxic phenotypes (C3 expression and secretion), morphological atrophy (volume, area and branching complexity) and synaptic dysfunction (synapse coverage), thereby improving neuronal plasticity impairment in DACI-model mice.

### High-glucose and cholesterol promotes pathological astrocytic responses through astrocytic SCAP-C3 signaling

**Fig. 7:**
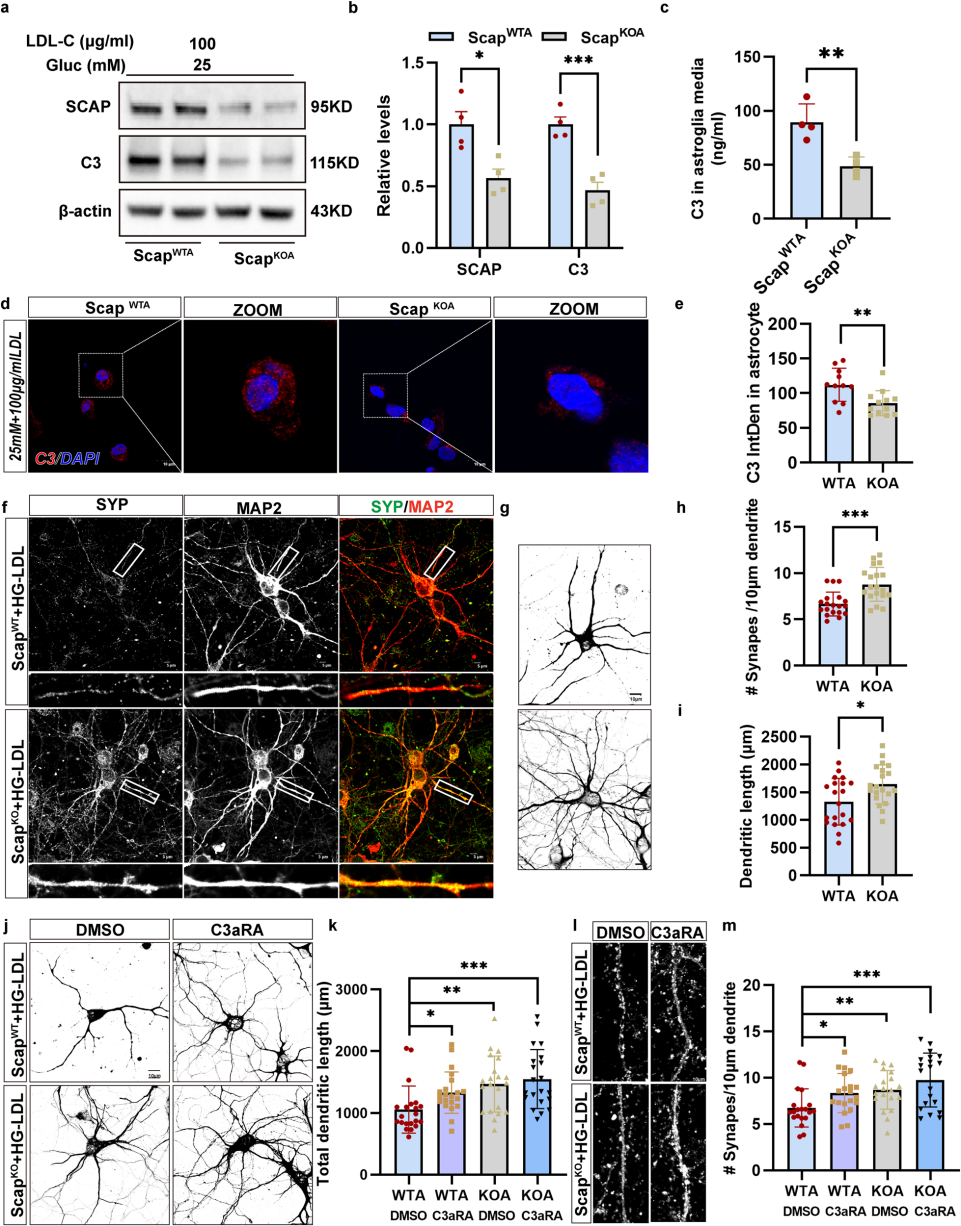
SCAP in astrocytes affects neuronal dendritic morphology and synaptic density via C3-C3aR binding in vitro. **a**, **b**The SCAP and C3 expression in astrocytes from wild-type (Scap^WTA^) and Aldh1l1-Cre-Scap^f/f^ (Scap^KOA^) mice treated with 25 mM glucose with LDL-C (100 μg/ml) (**a**) and the corresponding densitometric quantifications (SCAP and C3 normalized to β-actin) (**b**). β-actin was used as a control (*n* = 3 per group). **c** The ELISA of C3 levels in conditioned media of Scap^WTA^ or Scap^KOA^ treated with glucose (25 mM) and LDL-C (100 μg/ml). **d**, **e** The confocal images (**a**) and quantification (**b**) of C3 intensity in astrocytes (*n* = 10). **f** A double immunostaining of WT neurons with Syn and MAP2 cocultured with Scap^WTA^ or Scap^KOA^ treated with 25 mM glucose + 100 μg/ml LDL-C. **g** Representative dendritic structures of WT neurons cocultured with Scap^WTA^ or Scap^KOA^ treated with 25 mM glucose + 100 μg/ml LDL-C. **h**, **i** The quantification of synaptic density (**h**) and MAP2-positive dendritic lengths (**i**) in 25 mM glucose + 100 μg/ml LDL-C-treated Scap^WTA^ or Scap^KOA^ cocultured neurons. *n* = 20. **j**, **k** Representative images (**j**) and quantification of dendritic lengths (**k**) in MAP2-positive neurons treated with DMSO or C3aRA and cocultured with 25 mM glucose + 100 μg/ml LDL-C-treated Scap^WTA^ or Scap^KOA^. **l**, **m** Representative images (**l**) and quantification number (**m**) of SYP^+^ dendritic in neurons treated with DMSO or C3aRA and cocultured with glucose and LDL-C-treated Scap^WTA^ or Scap^KOA^. Scale bar, 10 μm. The data are means ± s.e.m., and the Student’s *t*-test (two-tailed) was performed for all the statistical analyses except **k** and **m**, where one-way ANOVA with Tukey’s multiple comparisons test was performed. *P < 0.05, **P < 0.01, ***P < 0.001, ****P < 0.0001.

To investigate astrocytic SCAP’s role in mediating C3 level increases, SCAP-KO astrocytes (KOA) were generated using the CRISPR‒Cas9 system and compared with WT astrocytes (WTA) from littermates. SCAP and C3 levels were significantly lower in KOA treated with 25 mM glucose and 100 μg/ml LDL-C than in WTA (Fig. 7a, b). Conditioned media from SCAP-KOA also showed reduced C3 secretion compared with WTA astrocytes after the high-glucose and LDL-C treatment (Fig. 7c). The immunofluorescence confirmed decreased C3 levels in SCAP-KOA (Fig. 7d, e). Under the high glucose and LDL-C treatment, the neurons cocultured with SCAP-KOA exhibited increased synaptic density (Fig. 7f, h) and increased axonal complexity (Fig. 7g, i) compared with neurons cocultured with WTA astrocytes, suggesting that the inhibition of SCAP could suppress complement C3 signaling and astrocyte neurotoxicity.

To clarify the role of SCAP in the responses of pathological astrocytes, we overexpressed SCAP in astrocytes. The expression of SCAP and complement C3 was increased by SCAP overexpression treatment (Supplementary Fig. 7a,b). At the same time, SCAP overexpression induced morphological changes and adopted a more stellate and reactive phenotype in contrast to their typical larger, flattened fibroblast-like morphology (Supplementary Fig. 7c). We then performed an astrocyte–neuron coculture (Supplementary Fig. 7d), and the neuron cocultured with SCAP-overexpressing astrocytes showed reduced dendrite length (MAP2) (Supplementary Fig. 7e) and reduced synaptic density (SYP) (Supplementary Fig. 7f). At the same time, we observed that with the overexpression of SCAP in astrocytes, the synaptic proteins PSD95 and GAP43 (Supplementary Fig. 7g) were reduced in neurons cocultured with them. Next, we performed SCAP rescue experiments to further verify the role of SCAP in the KO model. The data showed that SCAP overexpression rescued the reduction of SCAP and complement C3 in the KO model (Supplementary Fig. 7h,i), accompanied by the activation of astrocytic reactive morphology (Supplementary Fig. 7j), with a stellate appearance. Meanwhile, the dendrite length and synaptic density of neurons cocultured with SCAP-rescued astrocytes were reduced (Supplementary Fig. 7k–m), accompanied by a decrease in the level of synaptic proteins (PSD95 and GAP43) (Supplementary Fig. 7n). These findings indicate that SCAP activates the C3 signaling pathway in astrocytes to stimulate pathological astrocytic responses and neuroplasticity impairment.

To test whether C3aR mediates SCAP/C3 effects, neuron‒astrocyte cocultures were treated with a C3aR antagonist (C3aRA). The C3aRA preserved dendritic branching (Fig. 7j, top) and spine density (Fig. 7l, top) in WTA cultures, with no effect on neurons cultured with SCAP-KOA (Fig. 7j, l, bottom). The quantitative analysis confirmed that C3aRA treatment significantly increased the total dendritic length of WTA neurons compared with DMSO (Fig. 7k; *P* < 0.05), mimicking the enhanced dendritic complexity observed in KOA cocultures; no further increase was observed with C3aRA treatment in KOA cocultures. Similarly, the synapse density per 10 μm of dendrite was significantly elevated by C3aRA in WTA neurons (Fig. 7m; *P* < 0.05); no additional effect was observed in KOA cocultures. These findings suggest C3aRA mimics the protective effects of SCAP deletion, preventing neurotoxic astrocyte-induced neuronal changes.

Taken together, although the precise mechanism underlying SCAP-mediated C3 upregulation remains unclear, these results identify C3 as a SCAP target. Hyperglycemia, combined with cholesterol accumulation, drives SCAP-related C3 activation and release in astrocytes, mediating astrocyte‒neuron crosstalk through neuronal C3aR.

### Astrocytic SCAP mediates pathological astrocytic responses via the activating of NF-κB–C3 signaling

**Fig. 8:**
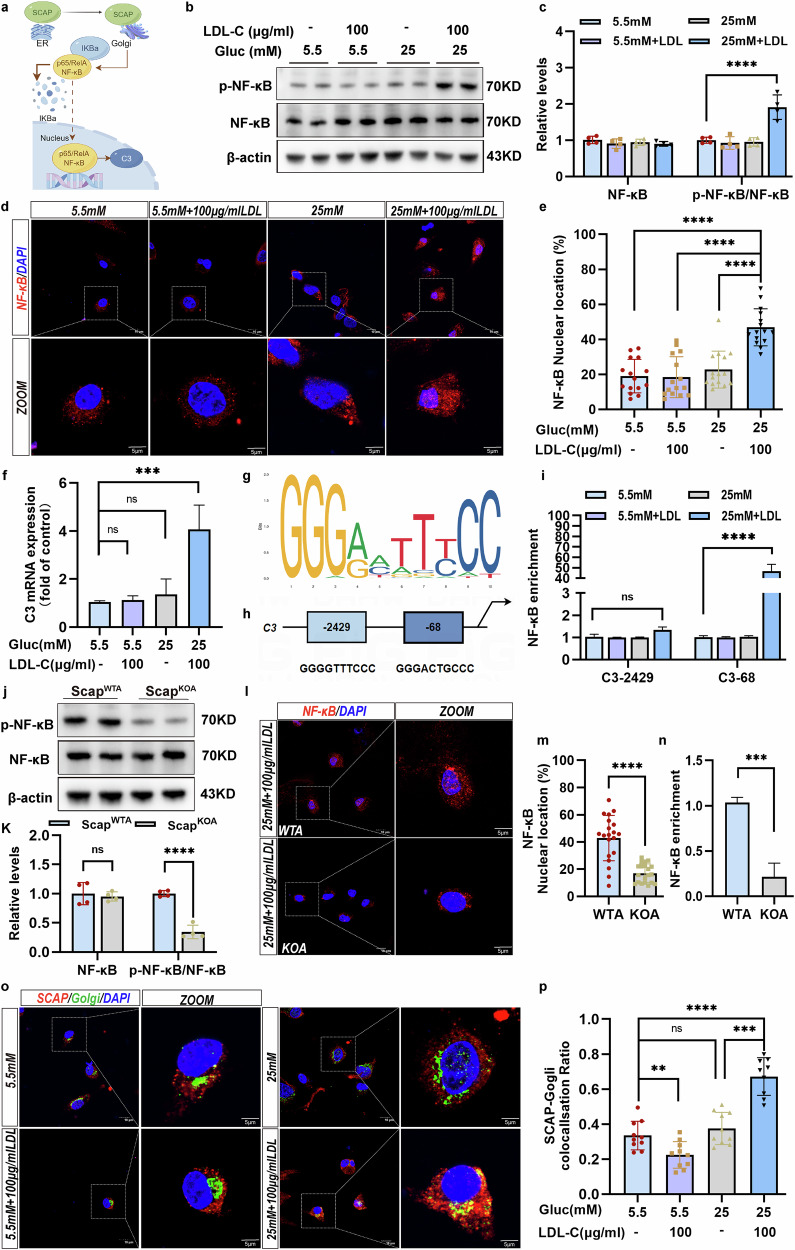
SCAP mediated the response of astrocytes to pathological conditions by activating NF-κB–C3 signaling. **a** A schematic diagram of the mechanisms by which SCAP activates NF-κB signaling. **b**, **c** The representative immunoblots (**b**) and the corresponding densitometric quantifications (**c**) of astrocytic NF-κB and p-NF-κB after glucose (Gluc) (5.5 mM or 25 mM) or (100 μg/ml) LDL-C treatment (*n* = 4 per group). **d**, **e** Representative confocal images (**d**) and the quantification (**e**) of NF-κB (red) and nuclei (blue) in astrocyte treated with Gluc (5.5 mM or 25 mM) or LDL-C (100 μg/ml). The bar graphs show the colocalization of NF-κB and nuclei in astrocytes (*n* = 15 per group). **f** A qPCR assay of C3 mRNA expression in astrocytes. **g**, **h** The NF-κB–C3 promoter binding motif (**g**) and the predicted NF-κB binding sites in the C3 promoter region (**h**). **i** The CUT&RUN–qPCR for NF-κB binding at C3 loci after Gluc and LDL-C treatment. **j**, **k** The representative immunoblots (**j**) and the quantification (**k**) of NF-κB and p-NF-κB in Scap^WTA^ and Scap^KOA^ with Gluc and LDL-C treatment. **l**, **m** Representative confocal images (**l**) and the quantification (**m**) showing NF-κB (red) and nuclei (blue) colocalization in Scap ^WTA^ and Scap^KOA^ (*n* = 20 per group). **n** A CUT&RUN–qPCR of the binding for NF-κB at C3 gene loci in Scap^WTA^ and Scap^KOA^ treated with Gluc and LDL-C. **o**, **p** The representative confocal images (**o**) and the quantification (**p**) of SCAP (red), Golgi 97 (green) and nuclei (blue) in WTA treated with Gluc (5.5 mM or 25 mM) or LDL-C (100 μg/ml). The bar graphs show the colocalization of SCAP and the Golgi in astrocytes (*n* = 10 per group). The data are expressed as the means ± s.e.m. The one-way ANOVA with Tukey’s multiple comparisons test was performed for all the statistical analyses except **k**, **m** and **n**, where the Student’s *t*-test (two-sided) was performed. *P < 0.05, **P < 0.01, ***P < 0.001, ****P < 0.0001. No other statistical comparisons are significant unless indicated.

NF-κB, a transcription factor, could regulates C3 gene transcription^38^ and contributes synaptic impairment through astrocytes process shortening^32^. Figure 8a presents a schematic illustration of the proposed mechanism by which SCAP activates the NF-κB signaling pathway, highlighting SCAP cycling between the ER and the Golgi apparatus, NF-κB activation and C3 transcription. To explore the role of astrocytic NF-κB in SCAP signaling, we analyzed the NF-κB activation in WT astrocytes treated with high glucose and cholesterol. The phosphorylated NF-κB levels increased significantly in astrocytes treated with 25 mM glucose and 100 μg/ml LDL-C, although neither treatment alone had this effect (Fig. 8b, c). The combined treatment also enhanced NF-κB nuclear translocation (Fig. 8d, e) and upregulated C3 mRNA expression (Fig. 8f). A CUT&RUN–Quantitative Real-time polymerase chain reaction (qRT-PCR) confirmed NF-κB binding to C3 genomic regions, with predicted NF-κB binding sites at the C3 promoter region (Fig. 8g–i). Meanwhile, the SCAP deletion in KO astrocytes significantly inhibited NF-κB phosphorylation (Fig. 8j, k), nuclear translocation (Fig. 8l, m) and binding to the C3 promoter region (Fig. 8n).

To clarify the role of SCAP in NF-κB/C3 signaling, we performed SCAP overexpression and rescue experiments. We found that SCAP overexpression and rescue mediated NF-κB activation, as demonstrated by increased NF-κB phosphorylation after SCAP overexpression and rescue in SCAP KO models (Supplementary Fig. 8a,c), as well as increased NF-κB nuclear translocation (Supplementary Fig. 8e–h). In addition, SCAP overexpression and rescue experiments mediated increased NF-κB transcriptional activity, also shown by the increased binding to the C3 locus (Supplementary Fig. 8b,d). HFD/STZ-induced mice exhibited elevated phosphorylated-NF-kappaB (p-NF-κB) levels compared with NCD-fed mice, which were reduced after astrocyte SCAP deletion (Supplementary Fig. 8i). The NF-κB inhibitor BAY11-7082 suppressed the SCAP-induced C3 activation in primary astrocytes, highlighting the SCAP–NF-κB–C3 pathway’s role (Supplementary Fig. 8j,k).

The immunofluorescence revealed that the SCAP translocation to the Golgi apparatus was significantly enhanced under combined high-glucose and cholesterol treatment but not by either treatment alone (Fig. 8o, p). SCAP-Golgi colocalization aligns with NF-κB activation and C3 transcription, suggesting that the high glucose disrupts the cholesterol-mediated ER retention of SCAP, facilitating SCAP translocation to the Golgi, which is essential for NF-κB–C3 signaling activation.

### SCAP recruits IκBα to the Golgi and activates NF-κB signaling

**Fig. 9:**
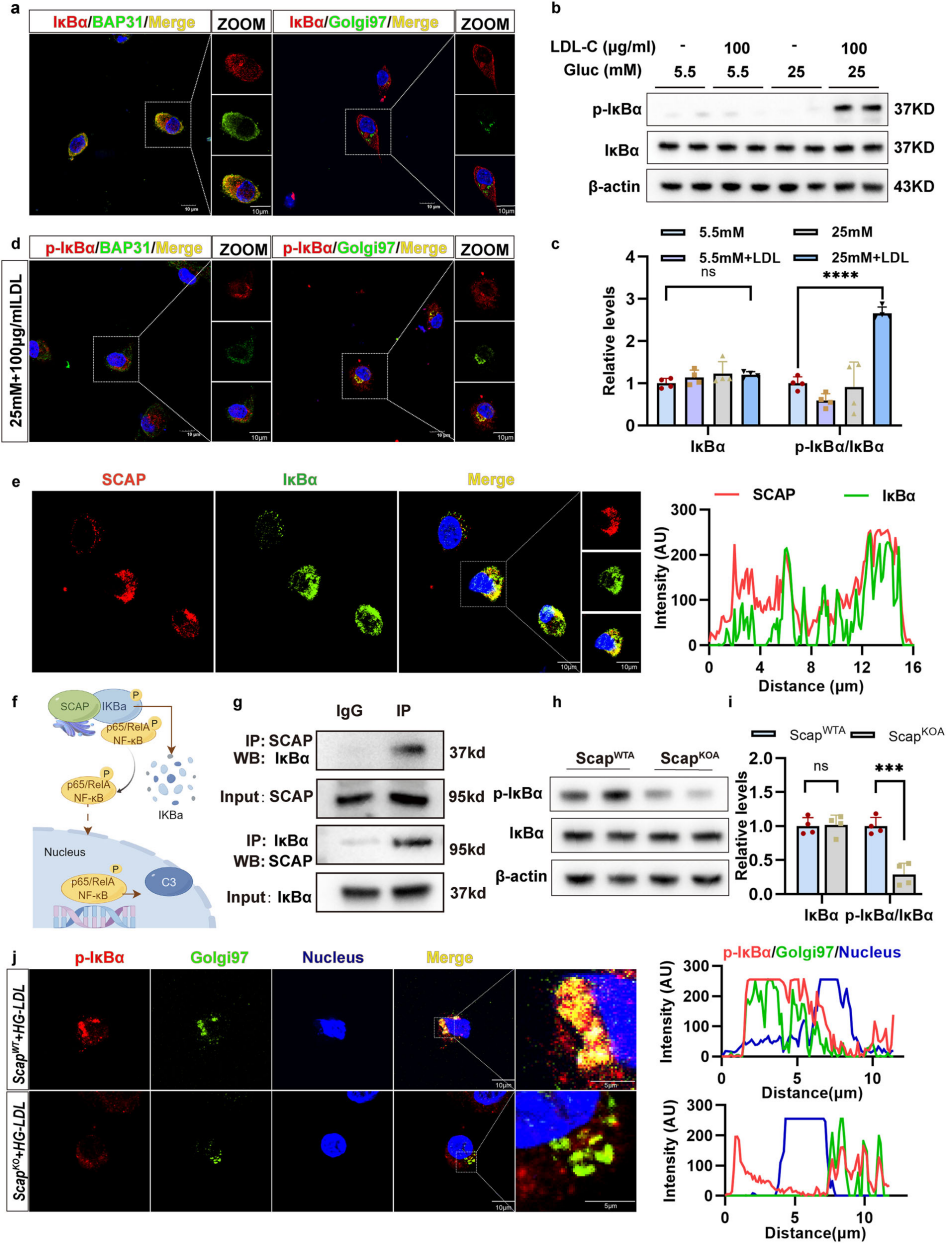
SCAP combined with IκBɑ-mediated Golgi apparatus translocation activates NF-κB. **a** The astrocytes were analyzed for colocalization of BAP31 and Golgi 97 with IκBɑ in the basal state via confocal microscopic imaging. Scale bars, 10 μm. **b**, **c** The representative immunoblots (**b**) and the quantification (**c**) of astrocytic IκBɑ and p-IκBɑ after treatment with different concentrations of glucose (Gluc) (5.5 mM or 25 mM) or (100 μg/ml) LDL-C (*n* = 4 per group per experiment). **d** The astrocytes were treated with Gluc (25 mM) and LDL-C (100 μg/ml), and the colocalization of BAP31 and Golgi 97 with p-IκBɑ was analyzed. Scale bars, 10 μm. **e** The immunofluorescence staining of SCAPs and IκBɑ in astrocytes. Scale bars, 10 μm. **f** A schematic diagram of the mechanisms by which astrocytic SCAP activates IκBɑ/NF-κB. **g** The immunoblotting for immunoprecipitation with anti-SCAP, anti-IκBɑ and anti-NF-κB antibodies in astrocytes (*n* = 3). **h**, **i** The representative immunoblots (**h**) and the quantification (**i**) of astrocytic IκBa and p-IκBɑ in WTA and KOA treated with Gluc (25 mM) and LDL-C (100 μg/ml) (*n* = 4 per group per experiment). **j** The SCAP-KOA and control astrocytes (WTA) were treated with Gluc (25 mM) and LDL-C (100 μg/ml), and the colocalization of Golgi 97 with p-IκBɑ was analyzed in KOA and WTA. The data are expressed as the means ± s.e.m. The one-way ANOVA with Tukey’s multiple comparisons test was performed for **c**, and Student’s *t*-test (two-sided) was performed for **i**. n.s., nonsignificant; *P < 0.05, **P < 0.01, ***P < 0.001, ****P < 0.0001.

To investigate whether SCAP regulates NF-κB activation through Golgi trafficking, we examined the subcellular colocalization between SCAP and IκBα, an inhibitory subunit of NF-κB. Under basal conditions, IκBα localized to the ER but not the Golgi apparatus (Fig. 9a). Combined high-glucose and LDL-C treatment increased p-IκBα levels (Fig. 9b, c), and p-IκBα was found in the Golgi but not the ER (Fig. 9d). Immunofluorescence confirmed SCAP colocalization with IκBα in astrocytes (Fig. 9e), whereas coimmunoprecipitation showed a strong interaction between SCAP and IκBα (Fig. 9g). These findings suggest that SCAP may recruit IκBɑ to the Golgi and facilitate NF-κB activation through Golgi translocation (Fig. 9f).

We further clarified the relationship between SCAP and IκBα phosphorylation. In SCAP-KOA, p-IκBα levels were reduced under combined high-glucose and LDL-C treatment (Fig. 9h, i), and p-IκBα expression in the Golgi was significantly decreased (Fig. 9j). Consistently, SCAP overexpression also mediated the increased phosphorylation levels of IκBα (Supplementary Fig. 9a), indicating that SCAP promotes the phosphorylation of IκBα, paving the way for NF-κB activation. To further elucidate the role of SCAP in IκBα/NF-κB signaling activation, we validated the role of SCAP in recruiting IκBα to the Golgi apparatus and triggering Golgi stress. We confirmed that the overexpression of SCAP increased the recruitment of IκBα to the Golgi region; the colocalization of IκBα and Golgi97 was significantly higher than that of the vector group via confocal microscopy (Supplementary Fig. 9b–d). Meanwhile, immunofluorescence staining showed that Golgi phosphoprotein 3 (GOLPH3; a Golgi stress marker) was upregulated in the SCAP overexpression group, accompanied by an increase in p-IκBα (Supplementary Fig. 9e–g). These results suggest that SCAP upregulation can induce Golgi stress and provide a possible spatial basis for IκBα/NF-κB activation.

### Complement C3 may predict cognitive impairment and shows a U-shaped correlation with LDL-C in patients with T2DM

In DACI mice, elevated levels of C3 in hippocampal astrocytes were associated with cognitive deficits. This C3 upregulation was driven by SCAP activation, which occurred only under combined hyperglycemia and hypercholesterolemia; neither glucose nor cholesterol alone was sufficient to induce SCAP expression. To assess the clinical relevance of these findings, we evaluated 202 patients with T2DM, examining serum metabolic parameters and assessing cognitive function using MoCA and MMSE. Among these individuals, 76 were diagnosed with MCI, whereas 126 exhibited normal cognitive function. The MCI group was older, had fewer years of education and included a higher proportion of females (Table 1). The MCI group demonstrated lower cognitive test scores (Supplementary Fig. 10a) and significantly higher serum C3 levels than controls (*P* = 0.016; Supplementary Fig. 10b). The partial correlation analysis revealed a negative association between serum C3 and MoCA scores (*r* = −0.222, *P* = 0.016; Supplementary Fig. 10c) and logic memory test (LMT) score (*r* = −0.151, *P* = 0.045) and a positively correlation with the auditory verbal learning test, delayed recall (AVLT DR) scores (*r* = 0.229, *P* = 0.013; Table 2), indicating that elevated C3 levels are associated with global cognitive decline, verbal learning and memory abilities and contextual memory impairment. No significant correlations were found with other cognitive domains.

**Table 1:** Demographic, clinical and cognitive characteristics of T2DM patients with or without MCI.

Variable	MCI (*n* = 76)	Non-MCI (*n* = 126)	*P* value
Age (years)	65.58 ± 8.57	58.02 ± 10.51	<0.001^a*^
Female (*n*, %)	33 (43.42)	32 (25.40)	0.008^c*^
Education (years)	10.32 ± 3.61	13.10 ± 3.21	<0.001^a*^
BMI (kg/m^2^)	24.16 (21.83–26.06)	24.77 (22.67–27.27)	0.107^b^
Diabetes duration (years)	11.59 ± 6.53	11.10 ± 6.83	0.618^a^
Hypertension duration (years)	5 (0–10)	5 (0–10)	0.493^b^
Smoking (*n*, %)	29 (38.16)	64 (50.79)	0.081^c^
Statins (*n*, %)	16 (21.62)	36 (30.25)	0.189^c^
Other antilipidemic drugs (*n*, %)	8 (10.81)	14 (11.76)	0.839^c^
HbA1c (%)	8.69 ± 1.75	8.53 ± 1.73	0.536^a^
FBG (mmol/l)	7.42 ± 2.38	8.46 ± 3.86	0.063^a*^
2h-PBG (mmol/l)	16.86 ± 5.18	16.09 ± 4.54	0.325^a^
TG (mmol/l)	1.23 (0.88–1.79)	1.40 (0.94–2.12)	0.142^b^
Total cholesterol (mmol/l)	4.37 ± 1.15	4.24 ± 1.06	0.454^a^
LDL-C (mmol/l)	2.30 (1.80–3.13)	2.46 (1.87–3.00)	0.751^b^
HDL-C (mmol/l)	1.04 (0.89–1.23)	0.98 (0.85–1.15)	0.086^b^
C3 (μg/ml)	686.00 ± 338.93	593.65 ± 200.70	0.016^a*^
MoCA	22.18 ± 3.09	27.71 ± 1.23	<0.001^a*^
DST	9.99 ± 3.16	12.31 ± 2.69	<0.001^a*^
VFT	15.86 ± 3.87	18.36 ± 4.01	<0.001^a*^
CDT	3 (2–4)	3 (3–4)	<0.001^b*^
TMTA	71.00 (62.00–88.00)	61.00 (51.50–78.50)	<0.001^b*^
TMTB	158.50 (123.75–205.75)	133.50 (110.50–160.00)	<0.001^b*^
AVLT IR	13.90 ± 4.96	15.94 ± 5.42	<0.001^a*^
AVLT DR	4.44 ± 3.63	6.42 ± 4.26	<0.001^a*^
LMT	4 (3–7)	6 (4–9)	<0.001^b*^
MMSE	27 (25–28)	29 (27–29)	<0.001^b*^

^*^P < 0.05.

The data are presented as *n* (%), mean ± s.d. or median (interquartile range) as appropriate.

^a^Student’s *t*-test.

^b^Mann–Whitney *U* test.

^c^*χ*^2^ test.

*BMI* body mass index, *HbA1c* glycosylated hemoglobin, *FBG* fasting blood glucose; 2 h-PBG, 2-h postmeal blood glucose, *HDL-C* high-density lipoprotein cholesterol, C3 complement C3, *DST,* digit span test, *VFT* verbal fluency test, *CDT* clock-drawing test, *TMTA* trail-making test A, *TMTB* trail-making test B, *AVLT* *IR* auditory verbal learning test, immediate recall, *AVLT* *DR* auditory verbal learning test, delayed recall.

**Table 2:** Partial correlations of serum C3 with neuropsychological tests and serum lipids in T2DM.

Control variables		Total		MCI	
		*r*	*P* value	*r*	*P* value
MoCA	Age, gender and educational attainment	−0.222	0.016^*^	−0.367	0.018^*^
MMSE		0.073	0.433	0.040	0.806
DST		0.100	0.281	0.045	0.781
VFT		−0.017	0.858	0.156	0.330
CDT		0.077	0.410	0.208	0.191
TMTA		−0.119	0.201	0.019	0.908
TMTB		−0.136	0.143	0.011	0.945
AVLT IR		0.018	0.844	−0.019	0.908
AVLT DR		0.229	0.013^*^	0.398	0.010^*^
LMT		−0.151	0.045^*^	−0.126	0.316
Total cholesterol		0.027	0.768	−0.160	0.318
LDL-C		0.012	0.894	−0.120	0.453

^*^P < 0.05 (partial correlations were used here).

We also explored the relationship between serum C3 and cholesterol levels (LDL-C, total cholesterol). A U-shaped relationship was observed between LDL-C and serum C3 concentrations (*P* = 0.047; Supplementary Fig. 10d), with a cut-off point of 2.535 mmol/l (Supplementary Table 1), but no correlation with total cholesterol levels was observed (Supplementary Table 2 and Supplementary Fig. 10e). The logistic regression analysis revealed that age, sex, education level, BMI and serum C3 levels were significantly associated with MCI occurrence (Table 3). The multivariate analysis showed that age, education level and serum C3 levels were key factors of cognitive impairment in patients with T2DM. These findings suggest that elevated C3 is closely related to diabetes-related cognitive impairment clinically and that both excessively high and low LDL-C are associated with elevated C3.

**Table 3:** Risk assessment of MCI in a simple and multivariable logistic regression model in T2DM.

	Univariate analysis			Multivariate analysis		
	*P* value	OR (95% CI)		*P* value	OR (95% CI)	
Age (years)	<0.001^*^	1.048	1.122	0.003^#^	1.021	1.102
Gender (female/male)	0.026^*^	1.080	3.437	0.791	0.555	2.164
Education (years)	<0.001^*^	0.754	0.911	0.005^#^	0.763	0.954
BMI (kg/m^2^)	0.033^*^	0.847	0.993	0.111	0.862	1.015
Diabetes duration (years)	0.616	0.969	1.055	–	–	–
Hypertension duration (years)	0.576	0.977	1.043	–	–	–
HbA1c (%)	0.534	0.891	1.250	–	–	–
FBG (mmol/l)	0.068	0.809	1.008	–	–	–
2h-PBG (mmol/l)	0.323	0.968	1.105	–	–	–
TG (mmol/l)	0.520	0.755	1.153	–	–	–
Total cholesterol (mmol/l)	0.452	0.848	1.447	–	–	–
LDL-C (mmol/l)	0.768	0.660	1.359	–	–	–
HDL-C (mmol/l)	0.265	0.689	3.864	–	–	–
C3 (μg/mL)	0.024^*^	1.000	1.003	0.028^#^	1.000	1.003

^*^P < 0.05 (simple logistic regression was used).

^#^*P* < 0.05 (multivariable logistic regression was used).

## Discussion

Cognitive impairment in DM often presents as reduced attention, memory, perception and executive function, leading to functional impairments and lower quality of life^46,47^. The key contributors include aberrant lipid metabolism, hyperglycemia and chronic neuroinflammation^8,48^. This Article demonstrates that elevated astrocytic SCAP expression is pivotal in astrocyte dysfunction and impaired neuroplasticity during DACI progression. The SCAP deletion in astrocytes increased astrocytic complexity in the CA3 and DG hippocampal regions, enhanced synaptic coverage, reduced astrocytic C3 levels, attenuated neuroplastic injury and improved cognitive outcomes. Mechanistically, SCAP-mediated Golgi translocation activates NF-κB, regulating neuron–glial crosstalk via the NF-κB–C3–C3aR pathway, thereby restoring neuroplasticity. These findings highlight the critical role of astrocytic dysfunction in neuronal synaptic plasticity in DACI, both in vivo and in vitro.

The majority of patients with T2DM are obese and prone to hypercholesterolemia^49^. In addition to contributing to nonalcoholic fatty liver disease, hyperlipidemia, hypertension and cardiovascular disease, impaired cholesterol metabolism is linked to cognitive decline^50–52^. The cholesterol accumulation exerts the progressive detrimental effects on cellular and tissue function. Consistent with studies linking abnormal cholesterol metabolism to cognitive decline in DM^14,15^, we observed cholesterol accumulation in DACI-model mice, which correlated with impaired learning and memory. These results suggest cholesterol, rather than TG accumulation, may drive DACI progression.

Hippocampal atrophy, a key feature of dementia and neuropsychiatric disorders, underscores the importance of hippocampal neuroplasticity in DACI pathophysiology^53–55^. The hippocampus is particularly vulnerable to functional and morphological changes induced by altered cholesterol metabolism^56–58^, and statins have shown benefits in memory and learning^59,60^. Our analysis confirms that the DG and CA3 regions, crucial for memory formation, are especially sensitive to chronic cholesterol accumulation, which may underlie the cognitive impairments in DACI-model mice^61^. The changes in dendritic spine morphology, such as reduced spine density and altered spine types, correlate with cognitive deficits^62,63^. The HFD/STZ-induced diabetic mice exhibited cognitive impairment with reduced neuronal complexity, fine spines and synaptic density, suggesting that the impaired neuroplasticity may contribute to learning and memory deficits. In addition, synaptic protein analysis further demonstrated decreased hippocampal synapse density, indicating the adverse effects of DACI on synaptic plasticity. These findings align with studies linking altered cholesterol exposure to impaired hippocampal synaptic plasticity^64,65^.

Despite evidence linking hippocampal neuroplasticity to cholesterol metabolism, the underlying mechanisms remain unclear. Astrocytes play integral roles in CNS function, from maintaining homeostasis to influencing synaptic plasticity^39,66^. As key components of the brain’s lipid metabolism network, astrocytes respond to microenvironmental changes, either promoting neuronal damage^36^ or exerting neuroprotective effects^67^. Astrocytes are the primary source of C3, a neurotoxic component that overactivates the complement cascade, impairing synaptic plasticity^68^. In this Article, we observed an increased C3 expression in hippocampal astrocytes of HFD/STZ-induced DACI-model mice, suggesting a neurotoxic phenotype. This finding aligns with studies in type I diabetes^62^ and AD models^69^. In astrocyte–neuron cocultures, high glucose and cholesterol triggered neurotoxic changes, including altered dendritic morphology and reduced synaptic density. Our results further highlight the link between cholesterol metabolism, astrocyte dysfunction and neurodegeneration, emphasizing the role of cholesterol exposure in driving astrocyte-mediated neurotoxicity^70–72^.

Astrocyte responses to pathological changes are multifaceted and highly context-dependent, encompassing reactive astrogliosis, atrophy and diverse functional modifications, which substantially impacts CNS homeostasis and neurological disease progression and prognosis^73^. For instance, Litvinchuk et al.^74^. observed increased astrocyte volume and decreased complexity in PS19 mice, which was reversed by C3aR KO. In AD models, Olabarria et al.^75^ found hippocampal astrocyte atrophy, with hypertrophic astrocytes surrounding plaques, highlighting the heterogeneity of astrocytic responses. Our Article revealed that in DACI-model mice, astrocytes in the CA3 and DG hippocampal regions showed reduced volume and surface area, increased soma volume and decreased complexity, suggesting an atrophic morphology linked to DACI. The linear regression analysis showed a negative correlation between hippocampal cholesterol content and astrocyte volume in diabetic mice, suggesting that cholesterol accumulation may drive these morphological changes.

Astrocyte atrophy results in decreased territory size and synaptic coverage, impairing synaptic plasticity and contributing to cognitive dysfunction. The evidence indicates that astrocyte atrophy and reduced synaptic coverage are key factors in cognitive disorders^76,77^. In our study, double-labeling with GFAP and SYP showed reduced SYP+ puncta and smaller puncta volume in DACI-model mice, indicating decreased astrocyte coverage of synapses, which is linked to impaired synaptic plasticity^78^. This reduced coverage may cause neurotransmitter spillover and neuronal hyperexcitability, often observed in neurodegeneration^79^. These findings, alongside evidence of neuroplasticity impairment, support the hypothesis that astrocyte dysfunction contributes to the learning and memory deficits associated with glucose and lipid metabolism disorders in DACI.

The astrocyte responses to pathological changes are complex, involving reactivity, atrophy and functional alterations. SCAP, a cholesterol sensitizer, bridges metabolism and inflammation and amplifies inflammatory mediator production in metabolic diseases, particularly in cholesterol homeostasis disorders^19,20^. In this Article, we confirmed SCAP activation in astrocytes in DM-model mice, with increases in SCAP levels in the DG and CA3 regions, where astrocytes showed atrophy. Linear regression revealed strong associations between astrocytic SCAP content, astrocytic territory volume, C3 levels and behavioral outcomes, supporting the role of SCAP as a nutrient sensor driving astrocyte responses and behavioral changes following a homeostasis imbalance. The regional differences in SCAP-related changes we observed may be explained by the intrinsic heterogeneity of astrocyte subsets, regional metabolic demands and differential sensitivity to diabetes-induced inflammatory and metabolic stressors^80^. Astrocytes in the DG and CA3 regions may be more sensitive and susceptible to metabolic and inflammatory stress responses, perhaps because they are involved in adult neurogenesis, active synaptic remodeling and higher basal neuronal plasticity^81,82^. These properties may render these regions particularly vulnerable to hyperglycemia stressors, such as cholesterol dysregulation, hyperglycemia-induced inflammation and SCAP-mediated signaling. Thus, SCAP upregulation in these regions may be a response to local cholesterol demand or toxic accumulation, thereby promoting astrocytes to enter a reactive state. At the same time, the CA1 region may not trigger a similar upregulation of SCAP due to a lower initial metabolic load or more efficient cholesterol homeostasis mechanisms. Notably, CA3 (and the adjacent DG) experiences greater lipid accumulation under hyperglycemia conditions than CA1^83^, which could exacerbate astrocytic SCAP accumulation and NF-κB activation, leading to higher C3 production in those cells. Given that the DG and CA3 regions play key roles in memory encoding, pattern separation and retrieval^84^, the regional increase in SCAP-C3 expression may have important functional implications. Increased C3 expression in astrocytes may trigger local complement activation, thereby driving pathological synaptic elimination and neuronal damage^62,85^, ultimately leading to cognitive deficits unique to DACI. Therefore, the DG and CA3 regions may be key sites that connect SCAP-driven pathological astrocytic responses to impaired cognitive function in patients with diabetes.

To future demonstrate the role of SCAP in DACI pathology, we generated astrocyte-specific SCAP-KO mice. Although no significant changes in blood glucose, body weight or lipid parameters were observed, the SCAP deficiency alleviated cognitive dysfunction and impaired neuroplasticity compared with SCAP fl/fl DM-model mice. The C3 levels were decreased in astrocytes and the hippocampi of DM-model mice with astrocytic SCAP deletion. In cocultures, SCAP-KO reduced C3 elevation induced by high glucose and LDL-C, abrogating the neuroplastic changes in neurons. C3 and C3aR activation are implicated in AD and tau pathology, with C3aR inhibition mitigating neuronal defects^74^. We found that the C3aR blockade mimicked the protective effect of SCAP downregulation, preventing synaptic injury in neurons exposed to high glucose and cholesterol.

Emerging evidence suggests astrocytes can adapt to lifestyle changes, offering clinical benefits^30,31^. We observed that knocking out SCAP in astrocytes increased astrocyte territory volume, dendritic complexity and synaptic coverage, similar to caloric-restriction-induced remodeling^33^. These changes were linked to improved synaptic plasticity and cognitive performance. Furthermore, SCAP knockdown increased synaptic density, correlating with enhanced astrocyte synaptic coverage. NF-κB activation, previously linked to astrocyte plasticity, was found to be abnormally activated in HFD/STZ-induced mice and inhibited by SCAP deletion. These findings suggest that NF-κB may be involved in SCAP-mediated astrocyte plasticity in DACI. Although the exact reasons for astrocyte complexity loss remain unclear, our data highlight the role of astrocyte changes in impaired hippocampal neuroplasticity in DACI. Future studies should focus on pre- and postsynaptic components to clarify this relationship and the underlying mechanisms.

SCAP plays a key role in metabolic diseases by regulating inflammatory signaling pathways linked to cholesterol disorders^19,20^, such as SCAP-mediated Golgi trafficking in NF-κB activation. In this Article, NF-κB phosphorylation and nuclear translocation, which thereby activates C3-C3aR, were observed in astrocytes exposed to high-glucose and cholesterol treatment. Our study is supported by prior work showing that SCAP’s movement from the ER to the Golgi is required for optimal inflammatory signaling. Notably, Guo et al. demonstrated that the ER-to-Golgi translocation of the SCAP–SREBP2 complex was required for the full activation of the NLRP3^18^. This implies that without Golgi translocation, the NF-κB-dependent ‘priming’ signal for inflammasome activation is blunted. More recently, Fei et al. reported that canonical NF-κB activation requires SCAP-mediated Golgi translocation, coordinating with S1P/S2P proteases to activate SREBP1 and inflammatory responses^86^. Consistently, a metabolic study noted that the loss of SCAP attenuates lipopolysaccharide -induced IκBα phosphorylation and p65 nuclear entry, indicating that SCAP is essential for NF-κB activation independent of its lipid metabolism role^87^. These studies corroborate our claim that SCAP actively interfaces with the NF-κB pathway, beyond cholesterol regulation.

Recent findings highlight that the Golgi apparatus, as a signaling hub for NF-κB, is essential for NF-κB–C3 activation^20,86^. The Golgi apparatus is increasingly recognized as a platform for immune signal^88^. For NF-κB, Golgi structural proteins can modulate the pathway that depleting golgin-97 (a Golgi tethering factor) causes uncontrolled IκBα degradation and NF-κB activation^89^. Moreover, GOLPH3 has been shown to potentiate Golgi stress and activate AKT/IKK, thereby enhancing NF-κB signaling in endotoxemia^90^. Conversely, reducing GOLPH3 alleviates Golgi stress and blunts NF-κB-driven inflammation. These reports echo the theme that Golgi stress can amplify NF-κB signaling, which is exactly what our SCAP results indicate. Our finding that SCAP triggers Golgi stress is in agreement with studies where induced Golgi stress leads to NF-κB activation as a cellular response. This mechanistic link between organelle stress and inflammation underlines the significance of our work: it connects SCAP (a cholesterol trafficking protein) with Golgi stress and NF-κB, providing a novel angle on how metabolic and stress signals converge on inflammation.

Complement proteins, including C1q and C3, are upregulated in AD brain tissues^36^ and cerebrospinal fluid^87,91^, where they are pivotal in neuroinflammation and synapse elimination^88,92^. In this Article, we found that serum C3 levels were elevated in patients with T2DM and were associated with decreased global cognitive ability (MoCA score), episodic memory ability (LMT score) and learning and memory ability (AVLT DR score). These findings align with our observation of elevated hippocampal C3 levels and impaired neuroplasticity in diabetic mice. In diabetic mice, elevated C3 in the DG and CA3 regions was associated with impaired neuroplasticity and cognitive decline. Combining the findings from animal models with clinical observations highlights its role in neuroinflammation and synaptic plasticity impairment, suggesting that C3 may play an important role in T2DM-related cognitive dysfunction. In addition, we found that a U-shaped correlation between LDL-C and C3 levels is notable, suggesting that both excessively low and high LDL-C levels may cause elevated C3 in diabetes, and the mechanism may lie in changes in lipid metabolism and immune regulation, thereby mediating the occurrence of complications such as cognitive impairment. Our previous study also found an inverted U-shaped correlation between LDL-C levels and cognitive function in patients with T2DM^14^, with similar U-shaped relationships observed in cardiovascular risk^89,93^. This implies that maintaining LDL-C levels within an optimal range could be crucial for controlling C3-associated neuroinflammation, which may affect treatment strategies focused solely on lowering LDL-C.

However, our Article has several limitations. First, although we observed regional differences, we have not yet elucidated the exact molecular basis that leads to astrocyte heterogeneity in hippocampal subregions. Further studies using single-cell transcriptomics or spatial transcriptomics approaches may help to clarify the molecular basis behind the regional differences in astrocytic responses. Secondly, the specific mechanism of abnormal astrocyte morphology and reduced complexity has not yet been clarified, and single-cell transcriptomics or spatial transcriptomics will be used to further clarify the specific molecular mechanism in the future. Third, our findings were primarily derived from male mouse models, which limits the generalizability of the conclusions, especially given the known sex differences in DACI prevalence. Therefore, future studies should include both sexes to determine whether similar regional patterns also occur in female models and whether sex-specific hormonal environments alter the impact of astrocytic SCAP accumulation on NF-κB–C3 signaling and cognitive outcomes in DACI. Finally, although we believe that DACI and AD share commonalities in C3 expression, direct comparative studies between DACI and AD animal models would be valuable to conclusively determine commonalities.

Our findings elucidate the role of SCAP in mediating astrocytic responses to diabetic pathology in DACI, from both morphological and molecular perspectives. The increased expression of SCAP in astrocytes of diabetic mice leads to astrocyte atrophy, which impairs synaptic plasticity and contributes to cognitive decline. SCAP activation exacerbates inflammation via the C3 pathway, further impairing neuroplasticity and resulting in learning and memory deficits. The underlying mechanism involves the SCAP-mediated activation of the IκBɑ–NF-κB–C3 pathway, highlighting the critical crosstalk between astrocytes and neurons. These findings suggest that targeting astrocytic SCAP expression could offer a novel therapeutic strategy for treating DACI. In summary, SCAP functions as a key regulator of astrocytic responses in DACI, promoting cognitive impairment through the activation of the IκBɑ–NF-κB–C3 pathway, and its inhibition may represent a promising approach to mitigate DACI-related cognitive deficits.

## Supplementary information

Supplementary Information

## Data Availability

The datasets used during and/or analyzed during the current study are available from the corresponding author upon reasonable request.
