# Supplementary material for: Cholesterol-driven pathological astrocytic responses in diabetes-associated cognitive impairment through astrocytic SCAP accumulation and NF-κB–C3 signaling modulation
Source: Exp Mol Med. 2025 Sep 29;57(9):2083–105. doi: 10.1038/s12276-025-01534-w (PMC12508041; doi:10.1038/s12276-025-01534-w)
Supplement: Supplementary file 1 — Supplementary Information [file 12276_2025_1534_MOESM1_ESM.docx]

**Supplementary Information**

**Materials and methods**

***Tamoxifen treatment***

Tamoxifen (Sigma‒Aldrich, T5648) was freshly prepared each time by dissolving it in corn oil to a concentration of 20 mg/mL on a shaker at 37°C in the dark for at least 12 h. At 10 weeks of age, all the mice in the experimental groups received one daily injection of tamoxifen (100 mg/kg, intraperitoneal) for five consecutive days.

***Primary neuron culture***

Primary neurons were prepared from postnatal day 0 (P0) pups via a protocol described previously [42] with the Worthington Papain Dissociation System (Worthington Biochemical, # LK003153). Briefly, P0 mouse cortices were dissected and collected in cold Earle’s balanced salt (EBSS). Cortices were then resuspended in 2.5 ml of warmed EBSS supplemented with papain (20 units/ml) and DNase (2000 units/ml). Following a 12 min incubation at 37°C, the cortices were triturated 10 times using a 5 ml Pasteur pipette and then 10 times using a 1 ml pipette to make a single-cell suspension. The samples were then passed through a 70 μm mesh filter to remove debris, and the filtrate was centrifuged (2000 × g for 5 min) to pellet the cells. The cells were then resuspended in 1.6 ml of suspension media [1.375 ml of EBSS, 150 μl of albumin-ovomucoid inhibitor (10 mg/ml in EBSS), and 75 μl of DNase (2000 units/ml)]. This solution was layered on top of a 2.5 ml solution of albumin-ovomucoid inhibitor (10 mg/ml in EBSS) to create a continuous density gradient, and the samples were centrifuged at 1000 rpm for 5 min. The supernatant was discarded, and pelleted neurons were collected in warm neurobasal medium (Invitrogen, 21103-049) supplemented with B27 (Invitrogen, 17504-044), 200 mM L-glutamine (Invitrogen, 25030-081) and 1% penicillin‒streptomycin. For the plating of primary neurons, dishes were precoated with poly-L-lysine (PDL) for 2 h at 37°C. The dishes were washed twice with ddH_2_O immediately before the neurons were plated. For the MAP2-SYP imaging experiments, the cells were plated in 24-well glass bottom dishes at a density of 3.5 × 10^5^ cells per well. For immunoblotting, the cells were plated at a density of 1 × 10^6^ cells per well in 6-well plates.

***Primary astrocyte culture***

C57BL/6J WT and AS cKO mouse pups at P2–3 days were prepared for culture of primary astrocytes. After anaesthesia, the brain tissues were removed and placed in DMEM (Invitrogen, 12800-017). Under a microscope, the meninges, cerebellum, and brainstem were removed with a precision instrument, and the hippocampus was minced with ophthalmic scissors. Then, 0.25% trypsin (2–3 mL) was added to digest the tissues at 37°C for 10 min, and the resulting cell suspension was filtered with 70 μm nylon mesh. The cells were counted and seeded into PDL-coated culture flasks containing DMEM, 10% FBS and 1% penicillin/streptomycin. After 7–9 days, the astrocytes were purified (190–200 rpm/min, 18–20 h, shaking table at 37°C) and seeded in cell culture plates for experimental treatment.

***Method details***

***Mouse model of T2DM***

In this study, 7-week-old male C57BL/6J mice were obtained from Changzhou Cavens Laboratory of Animal Science and Technology (Changzhou, China). A mouse model of T2DM was established via the consumption of HFD (Research Diets, D12492) and the administration of STZ (Sigma‒Aldrich, S0130; dissolved in 50 mM citric acid buffer, pH 4.5) [43]. Mice fed a normal chow diet (NCD, Research Diets, D12450B) were used as controls. After a one-week acclimation period, 8-week-old male C57BL/6J mice were randomly assigned to two groups and given a 4-week dietary treatment. One group was fed a HFD (60% calories from fat), whereas the other group was fed a NCD (10% of calories from fat). After 4 weeks on their respective diets, the mice fed a HFD were deprived of food for 12 h, followed by daily intraperitoneal injection of low-dose STZ (40 mg/kg) for 5 consecutive days at 12 weeks. The mice in the NCD group received the same volume of citric acid buffer. Nine days after the first STZ injection, blood samples were collected from the tail vein, and fasting blood glucose levels were detected using a glucometer (Yuwell, 580). The mouse model of DM was considered successfully established if HFD/STZ-induced diabetic mice had fasting blood glucose levels higher than 11.1 mmol/L in three consecutive tests [44]. These diabetic mice remained on their HFD for an additional 16 weeks. Cognitive performance was evaluated via behavioural tests in 28-week-old mice.

***Insulin tolerance test (ITT) and glucose tolerance test (GTT)***

After 8 h of fasting, the mice received an intraperitoneal injection of glucose solution (1 g/kg body weight) and were subjected to a GTT. Blood was collected from the tail, and glucose was measured at 0, 15, 30, 60, and 120 min after injection via a glucometer (Accu-Chek®, Roche, Basileia, Switzerland). For the ITT, the mice were fasted for 6 h and injected with insulin at a dose of 0.75 U/kg.

***Behavioural tests***

**Morris water maze (MWM)**

To evaluate spatial learning and memory, the MWM test was implemented according to previous publications. A platform (10 cm) was positioned 1 cm below the water’s surface and positioned in the southwest quadrant equidistant from the pool wall and pool centre. During the training phase, each individual mouse received consecutive trials for 5 continuous days, followed by a probe trial. On the probe trial day, the platform was removed, and the mice were allowed to swim for 60 s starting from the northeast quadrant, opposite to the quadrant where the platform was placed. Behavioural parameters were recorded by a video camera set on top of the circular pool, and the data were analysed via a digital tracking system (ANYmaze software, Stoelting).

**Y-maze test**

The Y-maze test was used to evaluate spatial working memory. The maze (30 cm × 6 cm × 15 cm) consisted of an isolated triangular central area and three identical arms at an angle of 120. The mice were allowed to explore the Y-maze for 8 min freely. If they could accurately remember the directions from which they came, the correct alternation order was ABC or another sequence of three nonrepeated arms. If they could not remember these directions, the alternation order would include at least two repeated arms (i.e., ABB, ABA, ACA, or ACC). Therefore, spontaneous alternation (%) was calculated via the following equation: spontaneous alternation (%) = correct alternation/(total arm entries–2) × 100. Moreover, the total number of arm entries and total distance parameters reflected autonomous movement ability. To ensure consistent and reliable experimental data, the surrounding environment was kept quiet, and the experiment was performed during the same period each day.

**Novel object recognition (NOR) test**

The NOR test was used to evaluate recognition memory ability. The mice were habituated to an open box (30 × 30 × 35 high cm) for 5 min on the day before the test. During the training session, two objects were placed in the open box, and the animals were allowed to explore them for 10 min. In the test session, the animals were placed into the same box, which contained one of the familiar objects and one novel object. The mice were then allowed to explore freely for 10 min, and the data were recorded with a video camera. The time spent exploring each object was automatically calculated with ANYmaze software (Stoelting). The preference index in the test session and the ratio of the amount of time spent exploring the novel object to the total time spent exploring both objects were analysed.

***Tissue perfusion and collection***

After the behavioural tests were conducted, the mice were anaesthetized, and blood was collected via cardiac puncture and left at 25°C for 1 h before centrifugation (3000 rpm, 20 min, 4°C). The plasma was subsequently obtained for the quantitative analysis of total cholesterol (TC), low-density lipoprotein (LDL) cholesterol and triglyceride (TG) levels or stored at − 80°C for further analysis. Afterwards, the mice were intracardially perfused with chilled 0.9% sodium chloride (Servicebio, G4702). The whole brains were harvested by decapitation, fixed in cold 4% PFA for 12 h, dehydrated in sucrose solutions at 30% at 4°C until they sank and cut into 40-μm-thick sections for histological analyses, such as immunofluorescence and filipin staining. For the qPCR and western blotting assays, fresh bilateral hippocampal tissues were collected after anaesthetization and stored at −80°C for further RNA isolation and total protein extraction. For the indicated experiments, at least 5 mice per group were sacrificed for brain or plasma collection.

***Biochemical analysis***

Serum samples and hippocampal tissues were collected from mice after sacrifice, and the levels of TG (Nanjing JianCheng, A110-1-1), TC (Nanjing JianCheng, A111-1-1) and LDL (Nanjing JianCheng, A113-1-1) were measured via enzymatic assays following the manufacturers’ instructions. The C3/C3a protein levels in the hippocampal tissue lysates were measured using a mouse C3 ELISA kit (Elabscience, E-EL-M0330) according to the manufacturer’s instructions.

***Immunofluorescence staining***

**Cells**

The cells seeded on coverslips were washed 3 times with PBS, followed by fixation in 4% PFA for 15 min at room temperature (RT). The cells were permeabilized with PBS containing 0.2% Triton X-100 for 15 min at RT, followed by blocking with 10% goat serum in permeabilization buffer for 30 min. After blocking, the cells were incubated with primary antibodies diluted in blocking buffer overnight at 4°C. The primary antibodies used included rabbit anti-MAP2 (Proteintech, 17490-1-AP, 1:500, 1.5 µg/ml), mouse anti-synaptophysin (Abcam, ab309493, 1:200, 1 µg/ml), mouse anti-GFAP (Sigma‒Aldrich, MAB360, 1:600, 1.67 µg/ml), rat anti-C3 (Novus Biologicals, NB200-540, 1:400, 2.5 µg/ml), rat anti-BAP31 (Thermo Fisher Scientific, MA3-002, 1:200, 2 µg/ml), mouse anti-Golgin-97 (Thermo Fisher Scientific, A-21270, 1:200, 1 µg/ml), mouse anti-GOLPH3 (Proteintech, 67777-1-lg, 1:400, 2.5 µg/ml), rabbit anti-SCAP (Thermo Fisher Scientific, PA5-115869, 1:500, 2 µg/ml), rabbit anti-NF-κB (Wanleibio, WL01980, 1:400, 2.5 µg/ml), rabbit anti-IκBα (Huabio, ET1603-6, 1:200, 2.5 µg/ml), and rabbit anti-phospho-IκBα (Huabio, ET1609-78, 1:200, 2.5 µg/ml). The cells were then washed with PBS and incubated with fluorescently labelled secondary antibodies diluted 1:1000 in blocking buffer for 1 h at RT. Alexa Fluor® 488-conjugated goat anti-rabbit IgG (ZSGB-BIO, ZF-0511, 1:200), Alexa Fluor® 594-conjugated goat anti-mouse IgG (ZSGB-BIO, ZF-0516, 1:200) and Cy3-conjugated AffiniPure goat anti-rat IgG (H+L) (Servicebio, GB21302, 1:200) were used. The cells were washed three times with PBS, and the nuclei were stained with DAPI (1:600, C1027, Beyotime) for 5 min at RT. Finally, the coverslips were mounted on microscope slides with Antifade Mounting Medium (Beyotime, P0126). Images were captured via a confocal microscope (OLYMPUS FV1000) under 60x and 100x objectives.

**Free-floating brain slices**

Fixed brains were embedded in optimal cutting temperature (OCT) compound, sliced into 40-µm-thick sections using a slicing microtome (Leica) and stored at –20°C in cryoprotectant solution (0.2 M PBS, 15% sucrose, and 33% ethylene glycol). The slices were washed with PBS and permeabilized with PBS containing 0.25% Triton X-100 for 60 min at RT, followed by blocking buffer (10% goat serum in permeabilization buffer) for 1 h at RT. The sections were then incubated with primary antibodies diluted in blocking buffer for 4 h at RT, followed by overnight incubation at 4°C. The primary antibodies used included mouse anti-GFAP (Sigma‒Aldrich, MAB360, 1:600, 1.67 µg/ml), rabbit anti-GFAP (Proteintech, 16825-1-AP, 1:200, 3.5 µg/ml), rat anti-C3 (Novus Biologicals, NB200-540, 1:400, 2.5 µg/ml), rabbit anti-SCAP (Thermo Fisher Scientific, PA5-115869, 1:500, 2 µg/ml), and mouse anti-synaptophysin (Abcam, ab309493, 1:200, 1 µg/ml) antibodies. The brain slices were then washed in PBS and further incubated with fluorescently labelled secondary antibodies (1:200 in blocking buffer) for 2 h at RT. After additional washes in PBS, the nuclei were stained with DAPI for 10 minutes at RT. Finally, the slices were mounted on microscope slides using Antifade Mounting Medium.

For free cholesterol (FC) detection, brain sections were stained with filipin complex (Sigma‒Aldrich, SAE0088, 1:100, 50 µg/ml) at RT for 30 min in the dark.

The same image exposure times and threshold settings were to analyse all the sections from the experimental groups. The analysis was performed by researchers that were blinded to the experimental groups.

***Quantitative analysis of immunohistological data***

Images were captured via a fluorescence microscope (OLYMPUS BX53, Japan) or a confocal microscope (OLYMPUS FV1000, Japan) under 60x and 20x objectives. The brain regions of interest (ROIs) were identified according to the cell densities marked by DAPI and by referencing the Allen Mouse Brain Atlas (Allen Institute for Brain Science, 2008). All the confocal stacks were acquired at a resolution of 1024 × 1024 pixels with a z-step of 0.5 μm. Two-dimensional images of C3/SCAP and GFAP+ astrocytes were created from maximum intensity projections of confocal z-stacks of images aligned in the x‒y plane and then analysed by ImageJ software (National Institutes of Health, USA). The morphology of GFAP^+^ astroglial cells in brain sections and cells was reconstructed into three-dimensional (3D) images with the surface-rendering feature of Imaris BitPlane 9.9 software (OXFORD Instruments, USA).

The number and integrated intensity of C3-positive or SCAP^+^ puncta in the ROIs were analysed via ImageJ software. C3-positive or SCAP ^+^ puncta colocalized with astrocytes were confirmed via three-dimensional projection images. To quantify the percentage of GFAP^+^ astrocytes that were also positively staining for C3 or SCAP, we used the following formula: C3^+^ or SCAP^+^ integrated fluorescence intensity/GFAP^+^ per astrocyte area × 100. The percentage of SCAP^+^ astrocytes in the hippocampal ROIs was defined as the proportion of SCAP^+^ and GFAP^+^ cells among all GFAP^+^ cells.

**Astrocyte morphology analysis**

To analyse the morphology of astrocytes in the ROIs, only GFAP^+^ cells with clearly visible cell bodies were included. The image background was subtracted from ImageJ, and astrocyte morphological features such as territory size (volume and area), soma volume, number of processes, total process length and number of process points were analysed via Bitplane Imaris software and Filaments option.

**Synaptic imaging and quantification**

For colocalization analysis and synaptic density measurement, Imaris Software Bitplane Spots and surface functions were used to analyse the 3D images. Briefly, brain tissue sections were coimmunostained with anti-synaptophysin and anti-GFAP antibodies and imaged using confocal microscope with a 60x oil immersion objective, 8x zoom and a 0.5 μm step. The image background was subtracted via ImageJ. The number of colocalized puncta was estimated using the Imaris software Bitplane Spots function. The volume of the reconstructed surfaces for GFAP^+^ and SYP^+^ cells was determined via Bitplane Imaris software and the Surface function, and the percentage of SYP^+^ GFAP^+^ astrocytes was calculated via the following formula: number of SYP^+^ GFAP^+^ cells /number of total GFAP^+^ cells.

***Golgi staining and morphometric analyses***

Golgi staining was conducted according to previously described methods [45]. Briefly, brains were freshly isolated and immersed in Golgi-Cox solution (5% w/v K2Cr2O7, 5% w/v HgCl2, and 5% K2CrO4) for one week at RT and then transferred to tissue-protectant solution (0.3% w/v NaH2PO4, 1% w/v Na2HPO4, and 1.8% w/v NaCl in 100 mM PBS) for 72 h at 4°C in the dark. The brains were subsequently sectioned at 100 μm thickness via a VT1200s Vibratome (Leica), followed by dehydration and clearing in xylene. Images of the dendrites were acquired via an optical microscope (Zeiss). For morphometric analysis, four to five individual neurons were selected randomly in tissue sections from each animal in all experimental groups and reconstructed via semimanual tracing via the ImageJ plugin NeuronJ. Sholl analysis was performed to measure the total dendritic length and the number of dendritic intersections with concentric circles positioned at radial intervals of 10 µm. Dendritic spine numbers were counted at lengths of 20 µm from the beginning of the secondary apical branches and the primary basal branches to obtain the average spine density, which was expressed as the number of spines/10 µm dendritic region.

***Cell isolation and magnetic-based cell sorting (MACS)***

The mice were perfused with ice-cold PBS, and adult mouse brains (without the olfactory bulb and cerebellum) were removed, rinsed in HBSS, minced and transferred to centrifuge tubes. Enzymatic dissociation was performed using an Adult Brain Dissociation Kit (Miltenyi Biotec, 130-107-667) following the manufacturer’s instructions. After enzymatic digestion, the sample was first passed through a 70 μm filter and then centrifuged following the establishment of a 30% Percoll gradient (2000 rpm, 12 min) to remove the myelin and remaining cell debris. The remaining cells were then centrifuged for 5 min at 300 × g at 4°C to pellet and resuspended in buffer containing 0.5% foetal bovine serum (FBS) in PBS. MACS was performed using different antibody-binding magnetic beads and a MACS Separator (Miltenyi Biotec, 130-042-201). For neuron magnetic labelling, the single-cell suspensions were first incubated with Nonneuronal Cell Biotin-Antibody Cocktail (Miltenyi Biotec, 130-115-389) (5 min, 4°C) and then incubated with AntiBiotin MicroBeads (10 min, 4°C) after being washed with buffer. Then, the cell suspension was applied to the column, and the flow-through containing unlabelled cells (neuronal cells) was collected. The isolation of unlabelled neuronal cells is achieved by depleting magnetically labelled cells. For the isolation of astrocytes, single-cell suspensions were incubated with ACSA-2 MicroBeads (Miltenyi Biotec, 130-097-678) (15 min, 4°C), and labelled ACSA-2^+^ cells (astrocytes) were collected via a MACS Separator. The sorted cells were pelleted, washed, and lysed for subsequent biochemical analysis.

***Cell treatment***

Astrocytes were seeded into either multiwell plates or glass coverslips coated with poly-d-lysine (PDL), depending on the experimental setup. After cell attachment, to mimic the glycolipid abnormalities observed in T2DM, the cell culture medium was replaced with DMEM containing 25 mM glucose and 100 μg/ml LDL-C (Sigma‒Aldrich, L8292) for 24 h. Control cells were cultured in DMEM containing 5.5 mM glucose, 25 mM glucose, and 100 μg/ml LDL-C.

To inhibit astrocytic SCAP, 4-hydroxytamoxifen (Sigma Aldrich, H6278, 500 μM in 2.5% ethanol in PBS) was diluted in DMEM to a concentration of 1 μM and was applied to primary astrocyte cultures, with the medium changed after 48 h. Coculture or cell lysis was performed after 5 days.

For overexpression of SCAP in astrocytes, 1 μg of SCAP overexpression plasmid DNA was used for 2×10^5 cells, and Lipofectamine^TM^ 3000 (Thermo Fisher Scientific, L3000015) was used as the liposome transfection reagent. The plasmid DNA and liposome transfection reagent were mixed and allowed to stand at room temperature for 15 min to allow the DNA-liposome complex to form completely. The above DNA-liposome complex (about 100 μL in total) was added dropwise to the target wells. After incubation for 6 h, the culture medium was replaced and cultured for another 48 h before the cells were used for subsequent experiments.

To evaluate C3aR signaling, primary neuron cultures were pretreated with 10 μM C3aRA (Calbiochem, #SB290157) for 1 h and then cocultured with astrocytes (see below). For each indicated in vitro experiment, at least three independent experiments were performed.

To inhibit astrocytic NF-κB signaling pathway, astrocytes were pre-treated with 2 μM Bay 11–7082 (HY-13453, MCE) for 6 h.

***Astrocyte-conditioned medium (ACM) collection and C3 ELISA***

After treatment with glucose or LDL, the astrocytes were cultured in DMEM for 72 h before ACM collection. The collected ACM was briefly centrifuged at 3000 × g for 10 min to remove cell debris. The supernatant was passed through a 0.22 mm filter and then concentrated via a concentrator (Millipore, 100 kDa, UFC9100) at 3000 × g for 40 min. The ACM concentration was measured with a BCA protein assay kit (KeyGEN, KGP902). C3 levels in ACM were determined using a mouse C3 ELISA kit (Elabscience, E-EL-M0330).

***Neuron‒astrocyte coculture***

For coculture, primary astrocytes from WT and AS cKO mouse pups were seeded on PDL-coated cell culture inserts (Costar, 3450 and 3470) and transferred at day 3 in vitro (DIV) for long-term astrocyte-neuron incubation. Immunostaining or western blot analysis was performed after 7 days of coculture.

***Protein extraction and western blotting (WB)***

Proteins were isolated from tissues and cell using ice-cold RIPA buffer (Fdbio Science, FD009) supplemented with protease and phosphatase inhibitors. The samples were subsequently centrifuged at 12000 rpm for 20 min at 4°C to collect the supernatant. Protein concentrations were determined via a BCA protein assay. Protein samples were diluted with 5x loading buffer. After boiling, equal amounts of total protein were loaded onto 4–20% FuturePAGE™ (ACE biotechnology, ET15420Gel), and transferred to a PVDF membrane (Millipore, USA). The membranes were then blocked with 5% milk in TBS/0.1% Tween-20 (TBST) and probed overnight at 4°C with the following diluted primary antibodies: rabbit anti-C3 (Proteintech, 21337-1-AP, 1:2000, 0.35 µg/ml), rabbit anti-C3aR (ABclonal, A6361, 1:2000, 0.5 µg/ml)**,** rabbit anti-SCAP (Abcam, ab190103, 1:1000, 0.4 µg/ml), mouse anti-GFAP (Sigma‒Aldrich, MAB360, 1:3000, 0.34 µg/ml), rabbit anti-PSD95 (Proteintech, 30255-1-AP, 1:1000, 0.45 µg/ml), mouse anti-synaptophysin (Abcam, ab309493, 1:1000, 0.2 µg/ml), rabbit anti-GAP43 (Abcam, ab75810, 1:1000, 0.224 µg/ml), mouse anti-NF-κB (Abcam, ab307840, 1:2000, 0.5 µg/ml), rabbit anti-p-NF-κB (Wanleibio, WL02169, 1:2000, 0.5 µg/ml), rabbit anti-IκBα (Huabio, ET1603-6, 1:2000, 0.5 µg/ml), rabbit anti-p-IκBα (Huabio, ET1609-78, 1:2000, 0.5 µg/ml), mouse anti-BAX (Proteintech, 60267-1-Ig, 1:2000 0.5 µg/ml), mouse anti-Caspase 3/P17/P19 (Proteintech, 66470-2-Ig, 1:3000 0.5 µg/ml), mouse anti-Bcl2 (Proteintech, 68103-1-Ig, 1:2000 0.5 µg/ml), and mouse anti-β-actin (Proteintech, 66009-1-Ig, 1:10000, 0.1 µg/ml). The membranes were washed 3×10 min in TBST and blotted with the following secondary antibodies: goat anti-rabbit-HRP (Proteintech, SA00001-2, 1:5000) and goat anti-mouse-HRP (Proteintech, SA00001-1, 1:5000). The membranes were again washed 3×10 min in TBST, incubated with enhanced chemiluminescence solution (Biosharp Life Science, BL520A), and visualized with a chemiluminescence imager system.

***Protein coimmunoprecipitation***

Astrocytes were cultured in T75 cell culture flasks. The cells were subsequently lysed with RIPA lysis buffer (Fdbio Science, FD008) containing a protease inhibitor for 20 min. The lysates were then centrifuged (12,000 rpm). 20 min). For subsequent immunoprecipitation, protein A/G magnetic beads (Biosharp Life Science, BL1226A) were incubated with 4 µg of IgG (Proteintech, 30000-0-AP), an anti-SCAP antibody (Abcam, ab190103) and an anti-IκBɑ antibody (Huabio, ET1603-6) at room temperature for 20 min. Then, the magnetic bead‒antibody mixture was incubated with the cell lysates (4°C, 8 h). After incubation, the beads were thoroughly rinsed three times with lysis buffer. Proteins were extracted from these beads, combined with protein loading buffer, and heated to 100°C for 8 min. The proteins were finally detected and analysed by western blotting.

***RNA extraction, reverse transcription, and qPCR***

Total RNA was extracted from cells, tissues or particles using TRIzol reagent (Vazyme, R401-01) according to the manufacturer’s instructions. Gene expression analysis was performed via reverse transcription‒quantitative PCR (RT‒qPCR), and detection of amplicons was based on SYBR Green dye-emitted fluorescence (Vazyme, Q341‒02). cDNA was synthesized using total RNA(300 ng to 1 μg) and HiScript III RT SuperMix (Vazyme, R223-01), and the reaction mixture diluted with UltraPure water to a final concentration of 1 ng/μl. Reactions were prepared in triplicate in 96-well plates (1 ng cDNA/well) and run on the Applied Biosystems StepOnePlus system (Thermo Fisher Scientific). The quantification of gene expression was based on the delta Ct method, which normalizes each gene of interest to the geometric mean of two or three housekeeping genes.

***CUT & RUN assay and analysis***

The CUT & RUN assay was conducted following the manufacturer’s protocol (Vazyme, HD101). Briefly, after treatment with different concentrations of glucose and LDL-C, the astrocytes were incubated with ConA Beads Pro at room temperature for 10 min, **a**nti-NF-κB antibody (ABclonal, A19653, 1:50) was added, and the mixture was rotated at room temperature for 2 h. Then, the mixture was washed twice, pG-MNase Enzyme was added, and the mixture was incubated at 4°C for 1 h. The mixture was subsequently washed twice, Cacl2 was added, and the mixture was incubated for 1 h on ice, stop buffer was added, and the mixture was incubated at 37°C for 30 min. DNA was extracted, and qPCR was used to detect C3 promoter sequence enrichment for NF-κB transcription factor-binding domains. The spikes in the DNA derived from the ƛDNA of *E. coli* were used for uniform correction.

**Supplementary Figure Legends**

**Supplementary Fig. 1.** HFD/STZ induces alterations in metabolic and serum biochemistry parameters. (a) Fluctuation of body weight. (b) Fast blood glucose (FBG) levels. (c-d) Intraperitoneal GTT levels (c) and intraperitoneal ITT levels (d) in NCD and HFD/STZ mice. (e-h) Levels of TG, TC, HDL cholesterol, and LDL in plasma. (i) Swimming speed for the MWM test. (j and k) Y-maze total arm entries (j) and total distance (k). (l and m) Recognition index (l) and representative traces (m) of NCD and HFD/STZ mice from NOR test. n = 8/group. Student’ s t-test (two-sided) was performed. All data are represented as mean ± SEM. *p < 0.05, **p < 0.01, ***p < 0.001, ****p < 0.0001, ns, not significant.

**Supplementary Fig. 2.** DACI conditions is not associated with changes in neuronal synaptic plasticity of CA1 and numbers of GFAP-Positive astrocytes. (a) Representative traces (left) and photomicrographs of the secondary apical branches (right) of pyramidal cells in the CA1 region. (b and c) Dendritic length (b) and dendritic spines/10 µm (c) in CA1 pyramidal neurons (n = 20/group). (d) Representative immunoblots of C3 and C3aR. (e) C3 protein levels in NCD and HFD/STZ mouse brains measured by ELISA (n=6/group). (f) C3 and GFAP co-immunostaining in the DG and CA1 regions of NCD and HFD/STZ mice. Scale bars: 50 µm. (g) Colocalization analysis of GFAP and C3 in astrocytes (Manders' coefficient), indicating the proportion of C3 signals colocalized with GFAP. The results showed that the colocalization degree of C3 and GFAP was significantly increased in the HFD/STZ group, **p = 0.0054 (n = 3/group). (h) Representative immunostaining of GFAP in the DG, CA3 and CA1 region of NCD and HFD/STZ mice. Scale bars: 50 µm. (i and j) Quantification of GFAP immunoreactivity (GFAP-IR) (i) and number of GFAP-IR cells (j) in the DG, CA3 and CA1 region of NCD and HFD/STZ mice. (k) Colocalization analysis of GFAP and SYP in astrocytes (Manders' coefficient) of NCD and HFD/STZ mice, indicating the proportion of SYP signals colocalized with GFAP. The results showed that the colocalization degree of SYP and GFAP was significantly reduced in the HFD/STZ group, **p = 0.0022 (n=3/group). Data is presented as mean ± SEM. Student’ s t-test (two-sided) was performed, *p<0.05; **p<0.01; ***p<0.001; ****P < 0.0001. ns, not significant.

**Supplementary Fig. 3.** Astrocytes subjected to high-glucose and LDL-C treatment altered neuron synaptic and dendritic morphology. (a) Schematic diagram of the coculture of primary astrocytes and neurons. (b) Double immunostaining of neurons cocultured with astrocytes treated with 5.5 mM glucose, 5.5 mM glucose + 100 µg/ml LDL-C, 25 mM glucose or 25 mM glucose + 100 µg/ml LDL-C with anti-synaptophysin (Syp) and anti-MAP2 (MAP2) antibodies. Scale bar, 5 µm. (c) Representative dendritic structure. Scale bar, 10 µm. (d) Quantification of total MAP2-positive dendritic lengths in the cocultured neurons in (b). n=20. (e) Quantification of the number of Syp^+^ synaptic puncta per 10 µm of dendrites (MAP2^+^) in the cocultured neurons in (b). n = 20. (f and g) C3 expression in astrocytes treated with low glucose (5.5 mM), high glucose (25 mM), or high glucose (25 mM) supplemented with LDL-C (100 µg/ml) was detected via western blotting. β-actin was used as a control. (n = 3/group/experiment). (h) ELISA quantification of C3 protein levels in the conditioned media of astrocytes treated with (f). (i and j) Representative confocal images of C3 (red) in astrocytes treated with different combinations of glucose (5.5 mM or 25 mM) or LDL-C (100 µg/ml). Bar graphs showing the average integrated fluorescence intensity (IntDen) of C3 in astrocytes. (n = 10). Scale bar, 10 µm. (k and l) Representative immunoblots of Bax, Bcl-2, and Cleaved Caspase-3 in primary astrocytes treated with 5.5 mM glucose or 25 mM glucose with LDL-C (n = 3/group). (*p = 0.0201 (Bax3), *p = 0.0138 (Bcl-2), **p = 0.0058 (Cleaved Caspase-3) between 5.5 mM glucose and 25 mM glucose with LDL-C). The data are expressed as the means ± SEM. One-way ANOVA with Tukey’s multiple comparisons test was performed for all the statistical analyses except (l), where student’ s t-test (two-sided) was performed. ns: nonsignificant; *P < 0.05, *P < 0.01, ***P < 0.001, ****P < 0.0001.

**Supplementary Fig. 4.** Astrocytic-specific knockout of SCAP via the Cre-loxP system. (a) Linear regression analysis between the immunodensity of ­filipin^+^ free cholesterol and the average astrocyte volume. Linear regressions are indicated by red (for NCD mice), yellow (for HFD/STZ mice) and black (both NCD mice and HFD/STZ mice are included) lines. (b) Colocalization analysis of GFAP and SCAP in astrocytes (Manders' coefficient), indicating the proportion of SCAP signals colocalized with GFAP in the hippocampal subregions of interest. The results showed that the colocalization degree of SCAP and GFAP was significantly increased in the HFD/STZ group (DG: **p = 0.007 (b1); CA3: **p = 0.0087 (b2); CA1: ns (b3) between the NCD and HFD/STZ groups) (n=3-4/group). (c) Colocalization analysis of GFAP and SCAP in astrocytes (Manders' coefficient), indicating the proportion of SCAP signals colocalized with GFAP in the hippocampus in the four groups (Cre- NCD, Cre- HFD/STZ, AS cKO NCD, and AS cKO HFD/STZ) (n=3-4/group). (d) The gene targeting strategy for generating. (e) Quantification of SCAP mRNA expression in primary astrocytes and neurons. (n = 3/group) (f) Quantification of SCAP mRNA expression in the liver, kidney and heart (n = 3/group). (g) Schematic illustration of the represents the chronological order of HFD feeding, STZ injection, cognitive testing using Aldh1l1-creERT2; SCAP-flox (AS cKO) mice. Data is presented as mean ± SEM. Student’ s t-test (two-sided) was performed, *p<0.05; **p<0.01; ***p<0.001; ****P < 0.0001.

**Supplementary Fig. 5.** Effect of astrocytic SCAP deletion on behavioral and metabolic indices in HFD/STZ induced DACI mice. (a) Fluctuation of body weight (n=6/group) (b) Fast blood glucose levels (n=9/group) (****p <0.0001 between Cre- NCD and Cre- HFD/STZ, ****p <0.0001 between AS cKO NCD and AS cKO HFD/STZ). (c) Intraperitoneal GTT levels in Cre- NCD, Cre- HFD/STZ, AS cKO NCD and AS cKO HFD/STZ mice (n = 6). (d-g) Levels of total TG, TC, HDL-C, and LDL-C in plasma of Cre- NCD, Cre- HFD/STZ, AS cKO NCD and AS cKO HFD/STZ mice (n = 10). (h) Quantification of dendritic complexity by Sholl analysis in four groups (Cre- NCD, Cre- HFD/STZ, AS cKO NCD, and AS cKO HFD/STZ). (i) Swimming speed for the MWM test (n=6/groups). (j and k) The platform crossover (j) and target quadrant retention time (%) (k) during the probe trial of the MWM test. (n = 6/groups). (l) Y-maze total arm entries (n = 6/group). (m) Y-maze alternation triplet (%) were increased at the Cre- HFD/STZ group compared to AS cKO HFD/TSZ group (n = 8/group) (**p = 0.004 between Cre- NCD and Cre- HFD/STZ) (*p = 0.0387 between Cre- HFD/STZ and AS cKO HFD/STZ). (n) NOR recognition index were increased at the Cre- HFD/STZ group compared to AS cKO HFD/STZ group (n = 6/group) (*p = 0.0191 between Cre- NCD and Cre- HFD/STZ) (*p = 0.0456 between Cre- HFD/STZ and AS cKO HFD/STZ). (o) Representative traces of Cre- NCD, Cre- HFD/STZ, AS cKO NCD and AS cKO HFD/STZ from NOR test. Data is presented as mean ± SEM. One-way ANOVA with Tukey’ s multiple comparisons test, *p<0.05; **p<0.01; ***p<0.001; ****P < 0.0001.

**Supplementary Fig. 6.** Astrocyte-specific SCAP ablation triggers a phenotypical switch in astrocytes of DG. (a) Representative immunoblots of C3 and C3aR (n = 3/group) (*p = 0.0278 (C3),*p = 0.0251 (C3aR) between Cre- NCD and Cre- HFD/STZ) (*p = 0.0137 (C3),*p = 0.0165 (C3aR) between Cre- HFD/STZ and AS cKO HFD/STZ). (b) C3 protein levels in Cre- NCD and Cre-/AS cKO HFD/STZ mouse brains measured by ELISA (n = 6/group). (c) Representative confocal images of GFAP- and C3-labeled astrocytes in the Cre- and AS cKO from NCD and HFD/STZ groups. Scale bars: 20 µm. (d) The average integrated fluorescence intensity (IntDen) of C3 colocalized with GFAP per astroglia in the hippocampus. (e) Colocalization analysis of GFAP and C3 in astrocytes (Manders' coefficient), indicating the proportion of C3 signals colocalized with GFAP in the hippocampus in the four groups (Cre- NCD, Cre- HFD/STZ, AS cKO NCD, and AS cKO HFD/STZ) (n=3/group). (f) Representative z-projection images of GFAP- and DAPI-labeled hippocampal subregions surveyed in the four groups of DG regin. The scale bar: 20 μm. Insets show higher magnifications (upper panel) and 3D rendering (lower panel) of boxed areas in the respective panels. Brain tissues were analyzed from five independent experiments (n = 5/group). (g and h) Quantification of astrocyte cell volume (g), and surface (h) in hippocampal subregions. n = 5/group, N = 4-5 cells/animal. (i) Colocalization analysis of GFAP and SYP in astrocytes (Manders' coefficient) of Cre- HFD/STZ and AS cKO HFD/STZ mice, indicating the proportion of SYP signals colocalized with GFAP. The results showed that the colocalization degree of SYP and GFAP was significantly increased in the AS cKO HFD/STZ group, *p = 0.0391 (n=3/group). One-way ANOVA with Tukey’s multiple comparisons test was performed for all the statistical analyses except (i), where student’ s t-test (two-sided) was performed. *p<0.05; **p<0.01; ***p<0.001; ****P < 0.0001. Data is presented as mean ± SEM.

**Supplementary Fig. 7**. SCAP in astrocytes mediates pathological astroglial responses. (a) Representative immunoblots of SCAP and C3 in SCAP Vector (VE-SCAP) and SCAP overexpression (OE-SCAP) astrocytes (n = 4/group). (b) Relative mRNA levels of SCAP in VE-SCAP and OE-SCAP astrocytes (n = 4/group). (c) Representative images of astrocyte morphology was examined by immunocytochemistry using a GFAP antibody (green) and nuclei were stained with DAPI (blue) in VE-SCAP and OE-SCAP astrocytes. Scale bar: 20 μm. (d) Double immunostaining of neurons with Syn and MAP2 cocultured with VE-SCAP or OE-SCAP astrocytes. Scale bar: 5 μm. (e and f) Quantification of MAP2-positive dendritic lengths (e) and Syp-positive synaptic density (f) in VE-SCAP or OE-SCAP astrocytes cocultured neurons (n = 10/group). (g) Representative immunoblots of the following synaptic proteins: PSD95 and GAP43 (n = 3/group). (h) Representative immunoblots of SCAP and C3 in *Aldh1l1-Cre-Scap^f/f^* (SCAP ^KO^) and SCAP overexpression *Aldh1l1-Cre-Scap^f/f^* (SCAP ^KO+OE^) astrocytes (n = 4/group). (i) Relative mRNA levels of SCAP in SCAP ^KO^ and SCAP ^KO+OE^ astrocytes (n = 4/group). (j) Representative images of astrocyte morphology using a GFAP antibody (green) in SCAP ^KO^ and SCAP ^KO+OE^ astrocytes. Scale bar: 20 μm. (k) Double immunostaining of neurons with Syn and MAP2 cocultured with SCAP ^KO^ or SCAP ^KO+OE^ astrocytes. Scale bar: 5 μm. (l and m) Quantification of dendritic lengths l) and synaptic density (m) in SCAP ^KO^ and SCAP ^KO+OE^ astrocytes cocultured neurons (n = 10/group). (n) Representative immunoblots of the following synaptic proteins: PSD95 and GAP43 (n = 3/group). Data is presented as mean ± SEM. Student’ s t-test (two-sided) was performed, **p<0.01; ***p<0.001.

**Supplementary Fig. 8**. Astrocytic SCAP mediated NF-κB activation. (a) Representative immunoblots of astrocytic NF-κB and p-NF-κB in VE-SCAP and OE-SCAP astrocytes (n = 3). (b) CUT&RUN-qPCR for NF-κB binding at C3 loci in VE-SCAP and OE-SCAP astrocytes. (c) Representative immunoblots of astrocytic NF-κB and p-NF-κB in SCAP ^KO^ or SCAP ^KO+OE^ astrocytes (n = 3). (d) CUT&RUN-qPCR for NF-κB binding at C3 loci in SCAP ^KO^ or SCAP ^KO+OE^ astrocytes. (e and f) Representative confocal images showing NF-κB (red) and nuclei (blue) colocalization in VE-SCAP and OE-SCAP astrocytes, and the colocalization of NF-κB with nuclei was analysed in VE-SCAP and OE-SCAP astrocytes. (g and h) Representative confocal images showing NF-κB (red) and nuclei (blue) colocalization in SCAP ^KO^ and SCAP ^KO+OE^ astrocytes, and the colocalization of NF-κB with nuclei was analysed in SCAP ^KO^ and SCAP ^KO+OE^ astrocytes. (i) Representative immunoblots of NF-κB and p-NF-κB in the four groups (Cre- NCD, Cre- HFD/STZ, AS cKO NCD, and AS cKO HFD/STZ). (n = 3/group). (j and k) Representative confocal images (j) of C3 (red) in WT astrocytes treated with DMSO or NF-κB inhibitor after treatment of HG and LDL-C. Scale bar: 10 μm. Bar graphs (k) showing the average integrated fluorescence intensity (IntDen) of C3 in astrocytes. Data is presented as mean ± SEM. Student’ s t-test (two-sided) was performed for (a-d, k), One-way ANOVA with Tukey’ s multiple comparisons test was performed for (I), *p<0.05; **p<0.01; ***p<0.001; ****P < 0.0001.

**Supplementary Fig. 9**. SCAP in astrocytes promoted Golgi recruitment of IκBɑ and Golgi stress. (a) Representative immunoblots of astrocytic IκBɑ and p-IκBɑ in VE-SCAP and OE-SCAP astrocytes. (b) Representative immunostaining of IκBɑ (green) and Golgi97 (marked Golgi apparatus, red) in primary VE-SCAP and OE-SCAP astrocytes (Scale bar = 10 μm or 5 μm). (c and d) The colocalization of IκBɑ with Golgi97 was analysed in VE-SCAP (c) and OE-SCAP (d) astrocytes. (e) Representative immunostaining of p-IκBɑ (green) and GOLPH3 (Golgi stress markers, red) in primary VE-SCAP and OE-SCAP astrocytes (Scale bar = 10 μm or 5 μm). (f and g) The colocalization of p-IκBɑ with GOLPH3 was analysed in VE-SCAP (f) and OE-SCAP (g) astrocytes.

**Supplementary Fig. 10.** C3 may be a biomarker to predict the progression of DACI in humans. (a) MoCA scores of T2DM patients with MCI (n = 76) and without MCI (n = 126) (****p < 0.0001). (b) Serum levels of complement C3 in T2DM patients with MCI and without MCI (*p = 0.016). (c) Correlations between the MoCA score and serum C3 levels in T2DM patients with MCI and without MCI. Correlations were found via linear regression, with r = −0.222 and p = 0.016. (d) Comparison of linear and U curve associations between LDL-C and C3. The cut-off point for LDL-C was 2.535 mmol/l. (e) Comparison of linear and U curve associations between total cholesterol and C3. The values are presented as the means ± SEM. Two-tailed Student’s t-tests were performed in a, b. *p<0.05; ****P < 0.0001.

**Supplementary table 1:** Comparison of line fitting and U-shaped curve fitting assessing the association between LDL-C and C3.

|  | R2 | *P* | A | B1 | B2 |
| --- | --- | --- | --- | --- | --- |
| Line | 0.001 | 0.782 | 626.569 | -7.038 |  |
| U-shape | 0.045 | 0.047* | 1011.494 | -350.033 | 69.026 |

**P*<0.05

Abbreviations: LDL-C, low density lipoprotein cholesterol; C3, Complement 3;

**Supplementary table 2:** Comparison of line fitting and U-shaped curve fitting assessing the association between TC and C3.

|  | R2 | *P* | A | B1 | B2 |
| --- | --- | --- | --- | --- | --- |
| Line | 0.006 | 0.270 | 540.511 | 18.994 |  |
| U-shape | 0.0407 | 0.490 | 459.084 | 60.368 | -4.902 |

**P*<0.05

Abbreviations: TC, Total cholesterol; C3, Complement C3;


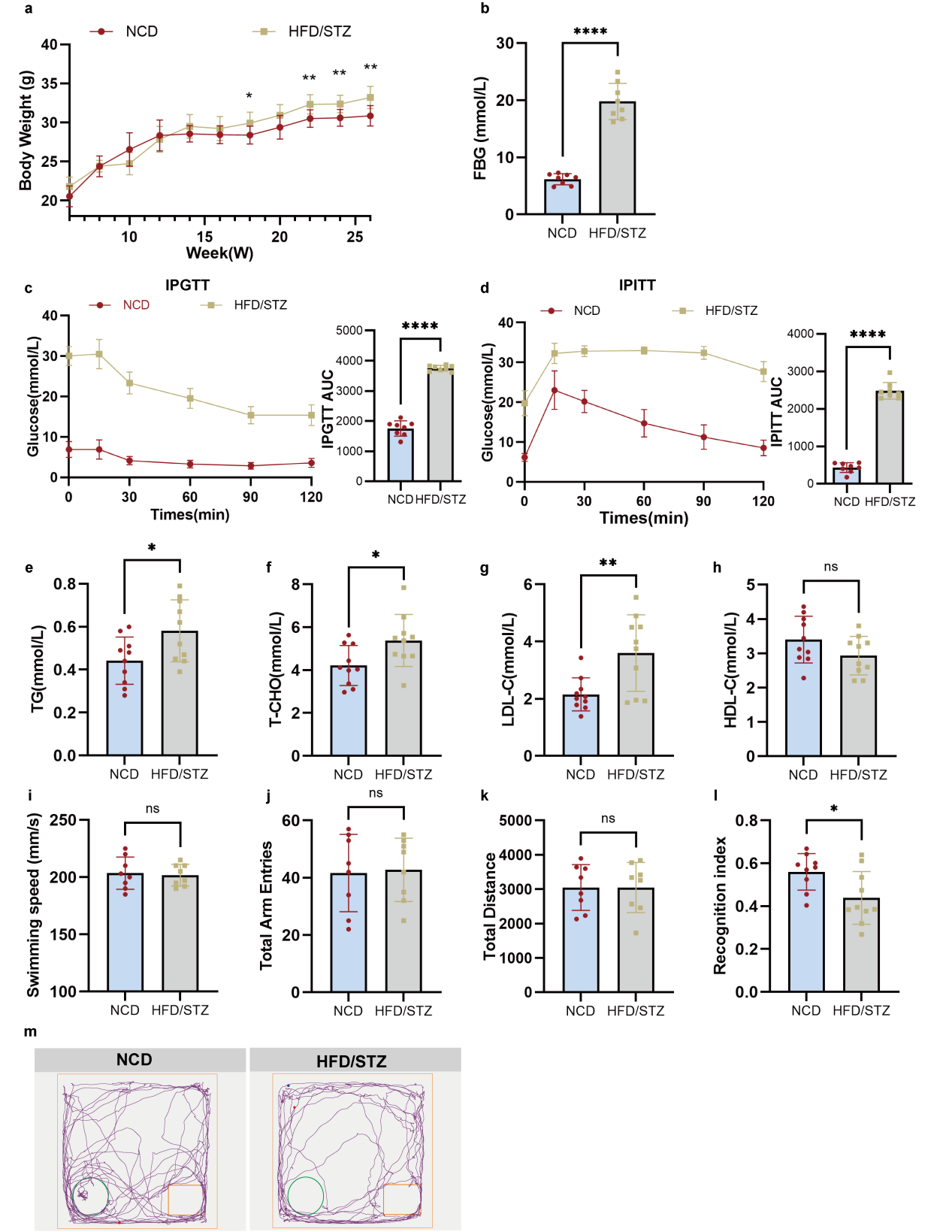


**Supplementary Fig. 1**


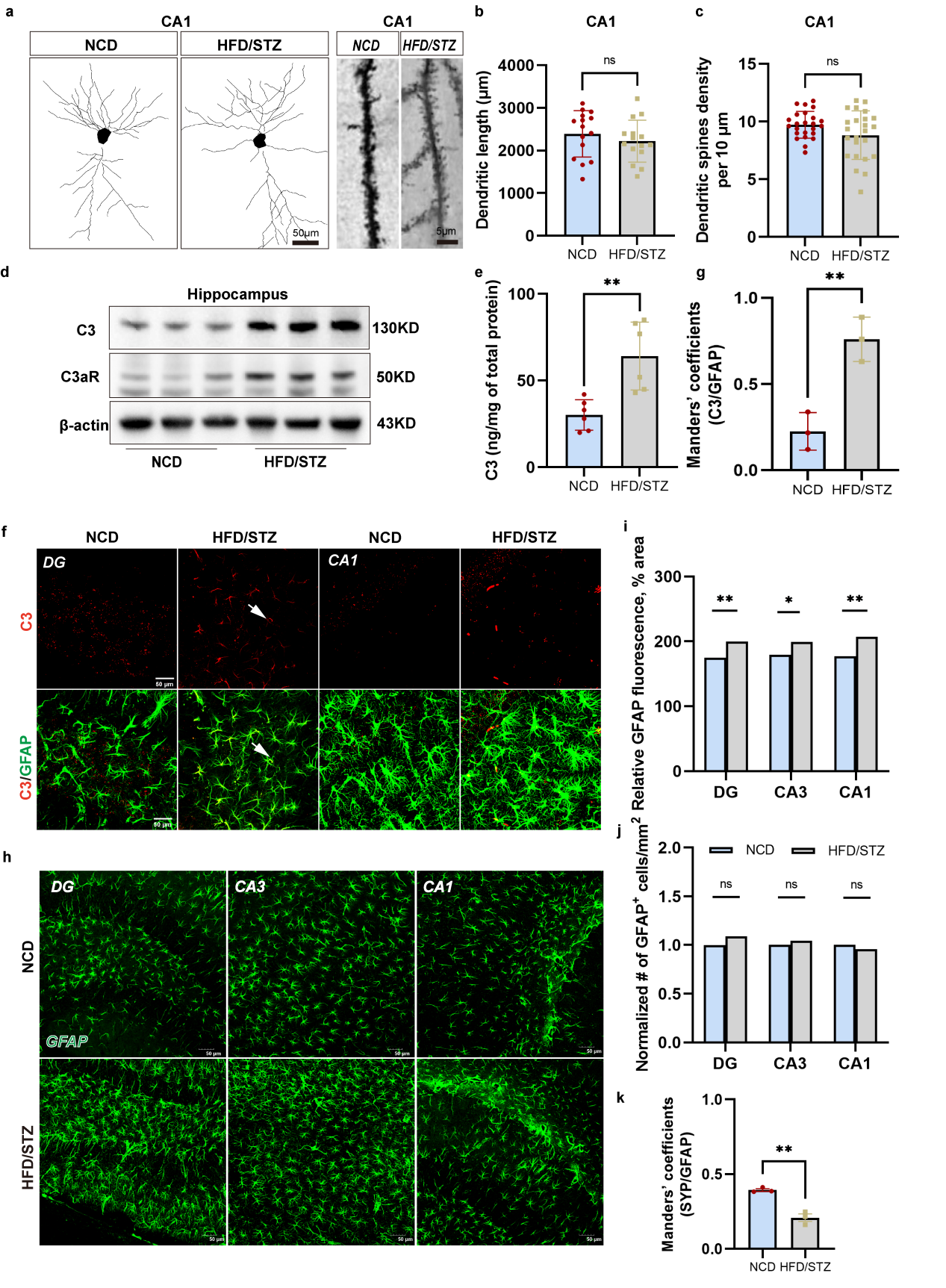


**Supplementary Fig. 2**

**
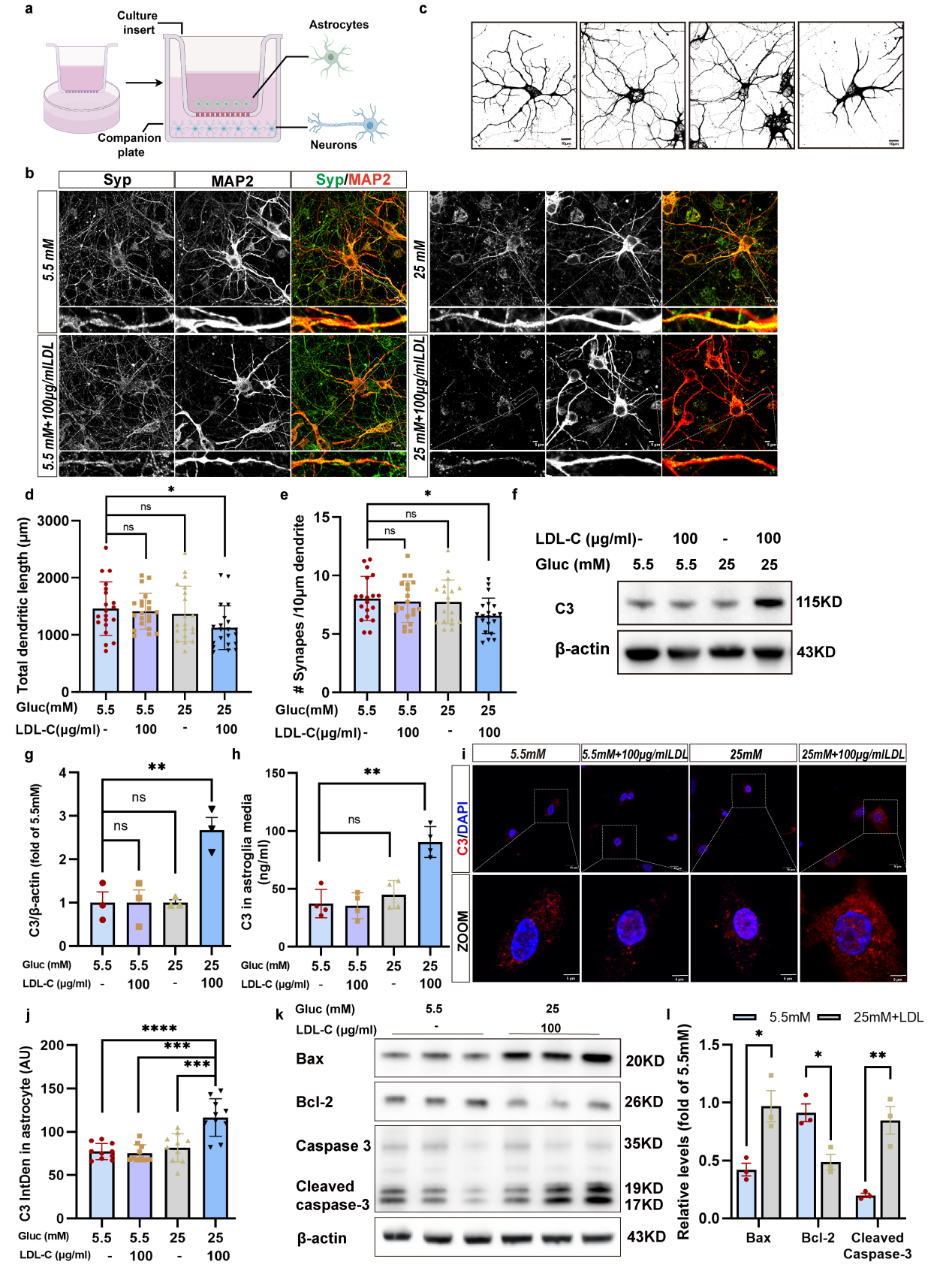
**

**Supplementary Fig. 3**

**
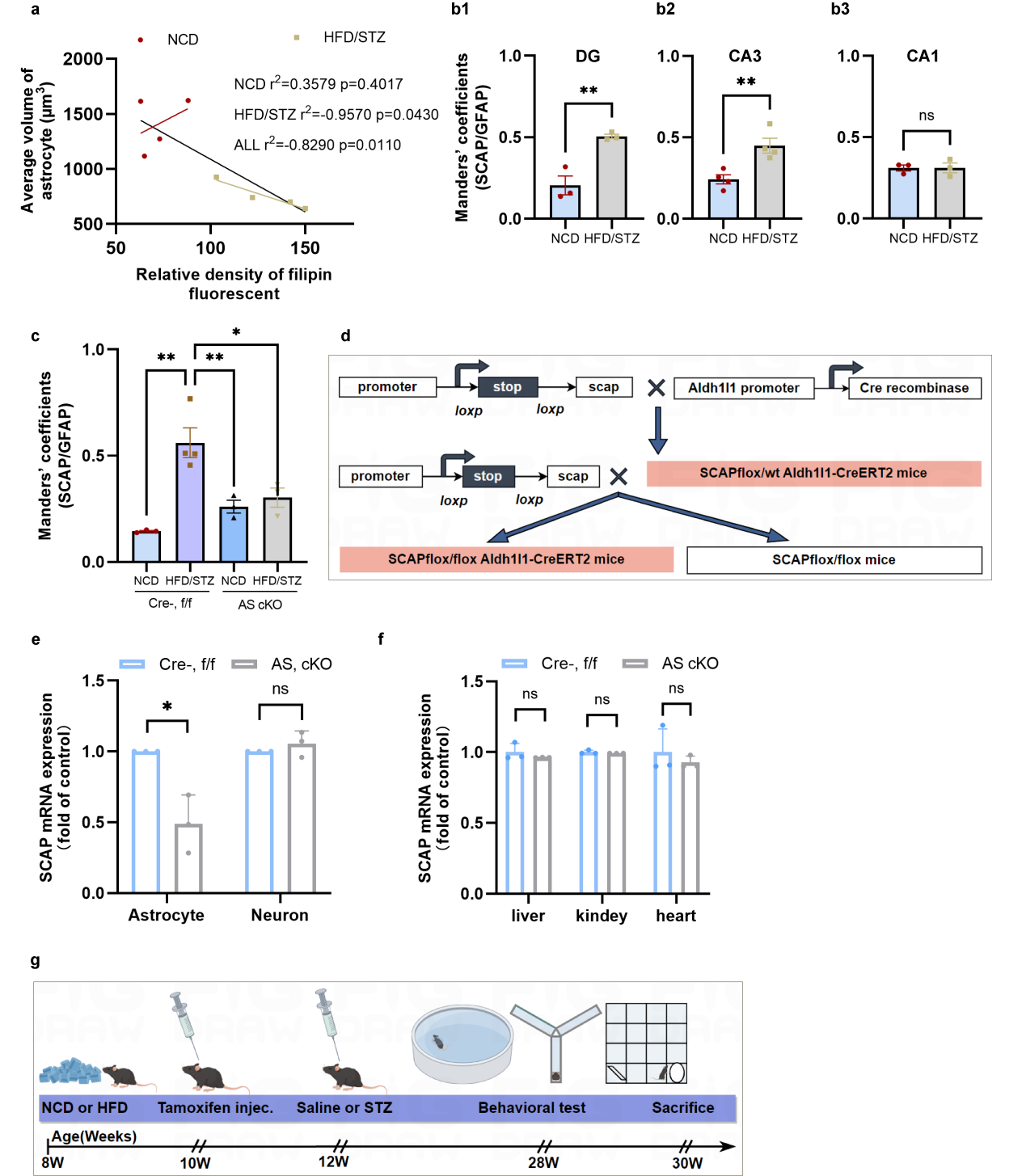
**

**Supplementary Fig. 4**

**
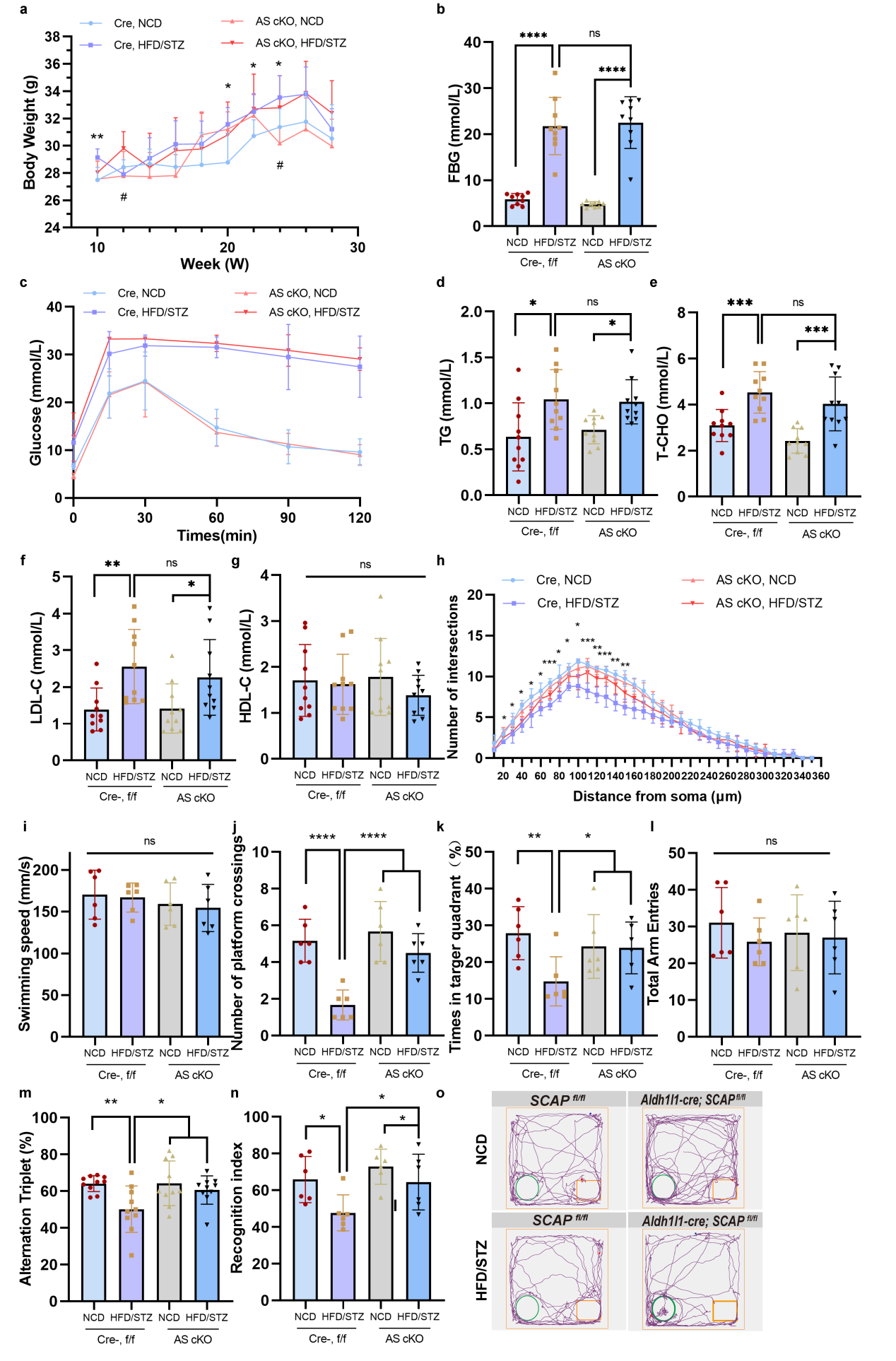
**

**Supplementary Fig. 5**

**
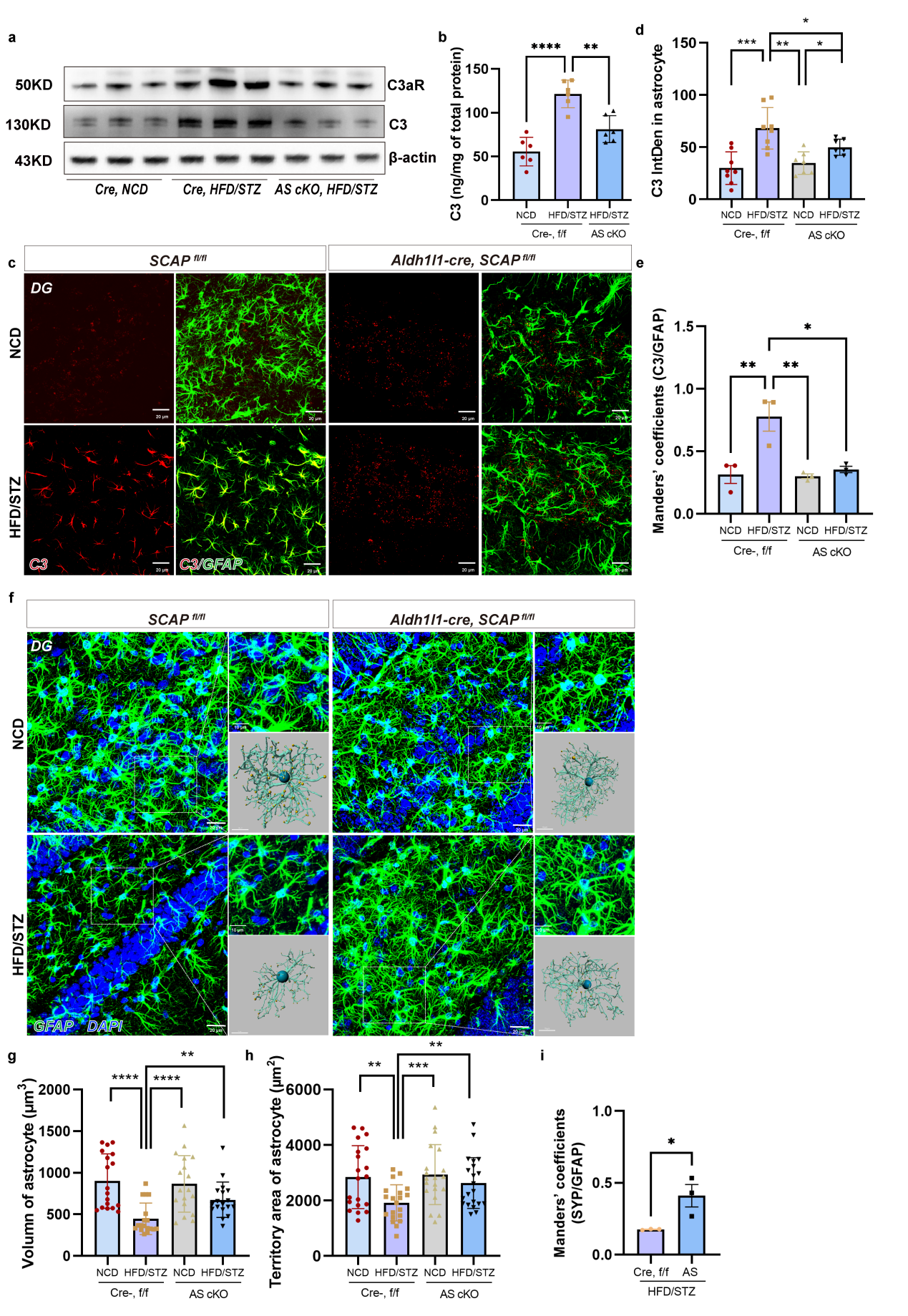
**

**Supplementary Fig. 6**


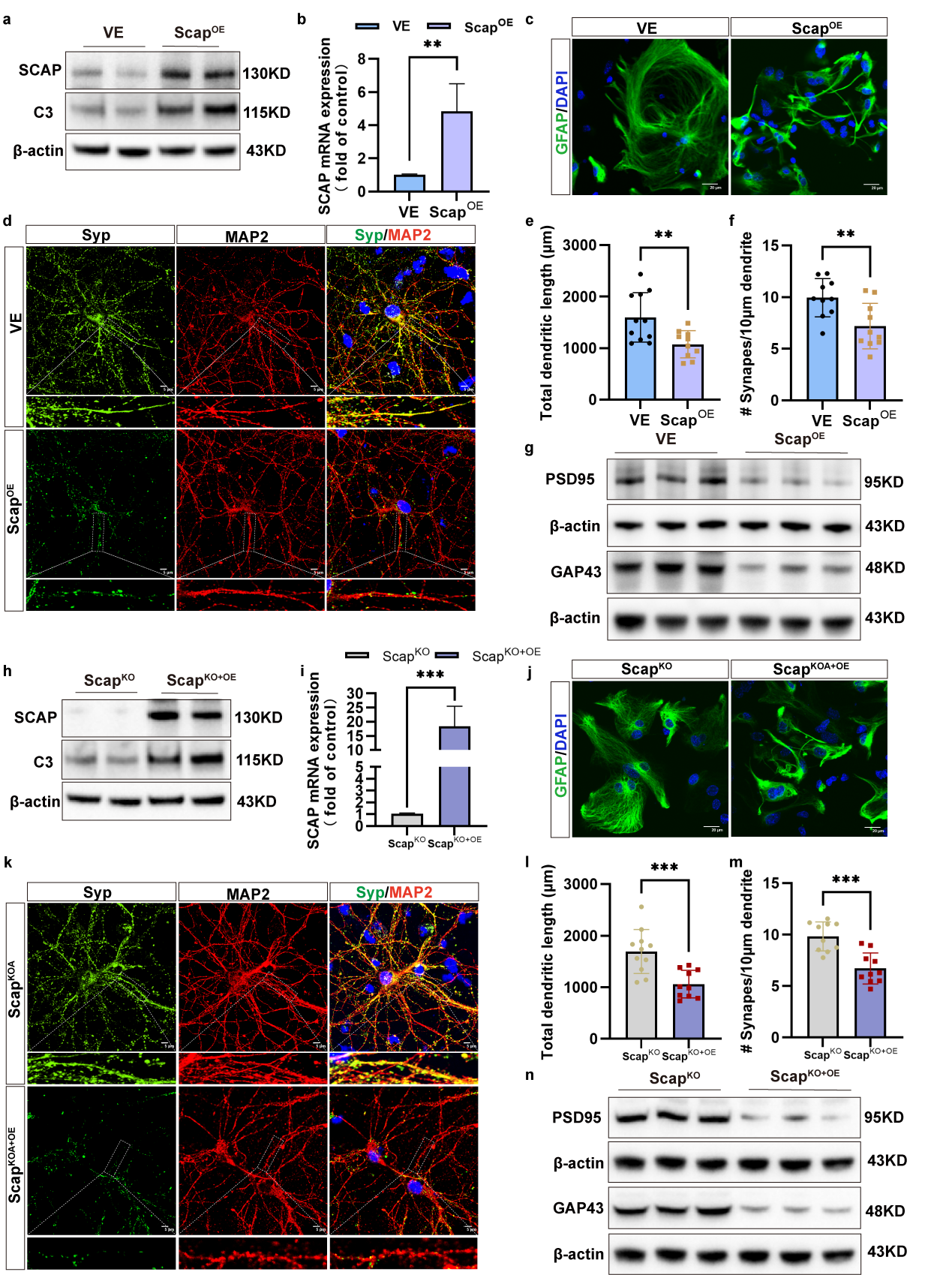


**Supplementary Fig. 7**

**
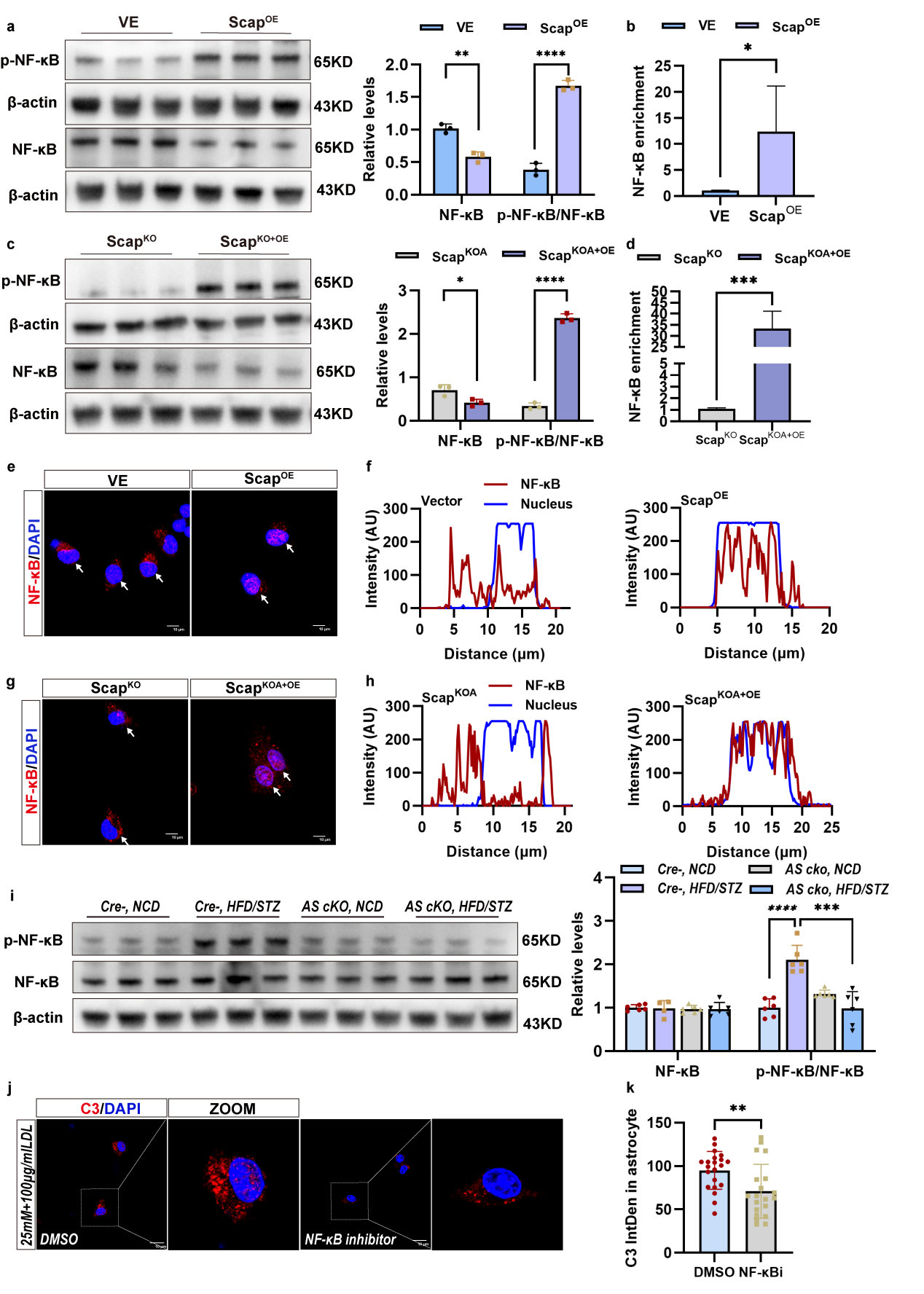
**

**Supplementary Fig. 8**

**
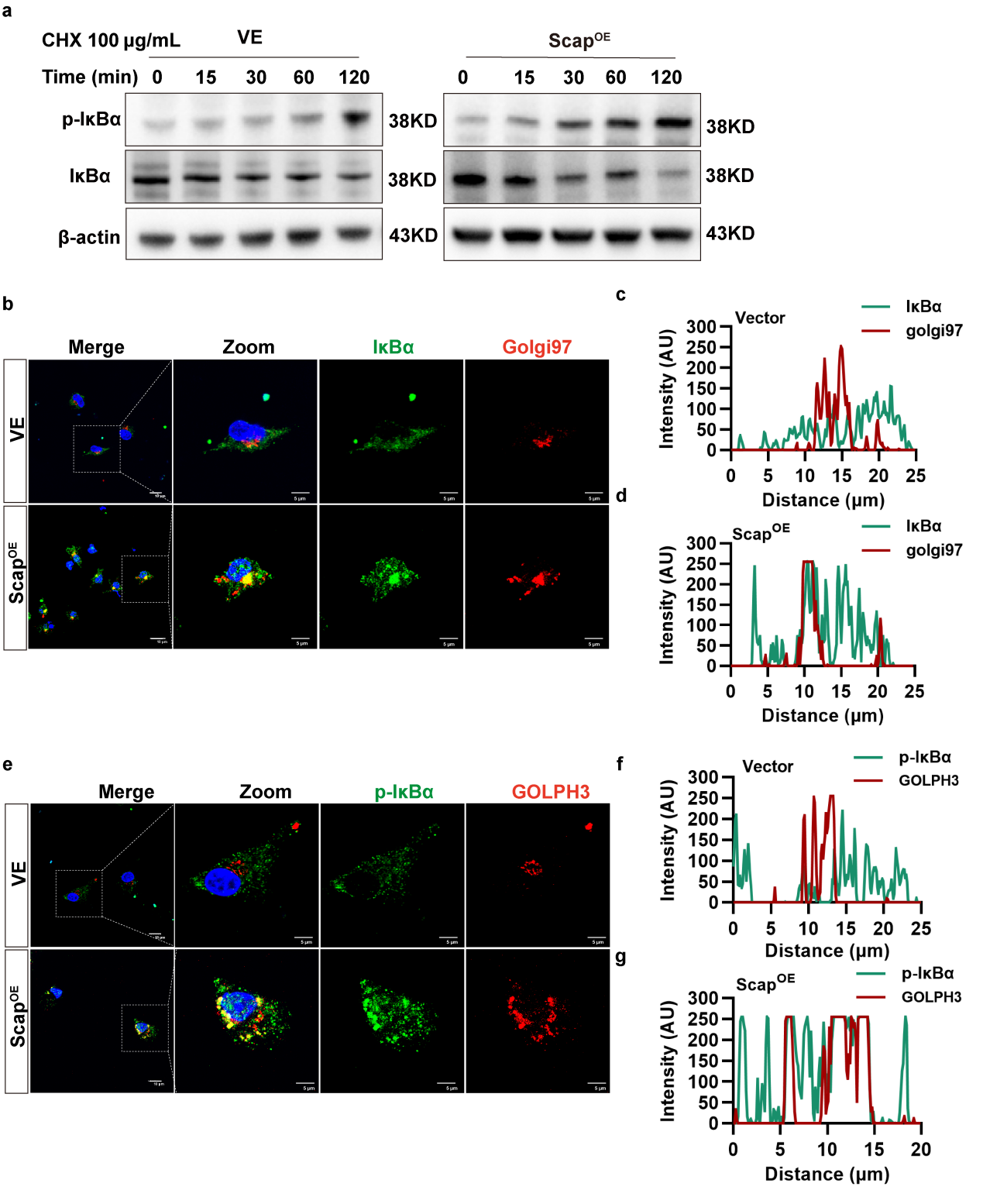
**

**Supplementary Fig. 9**

**
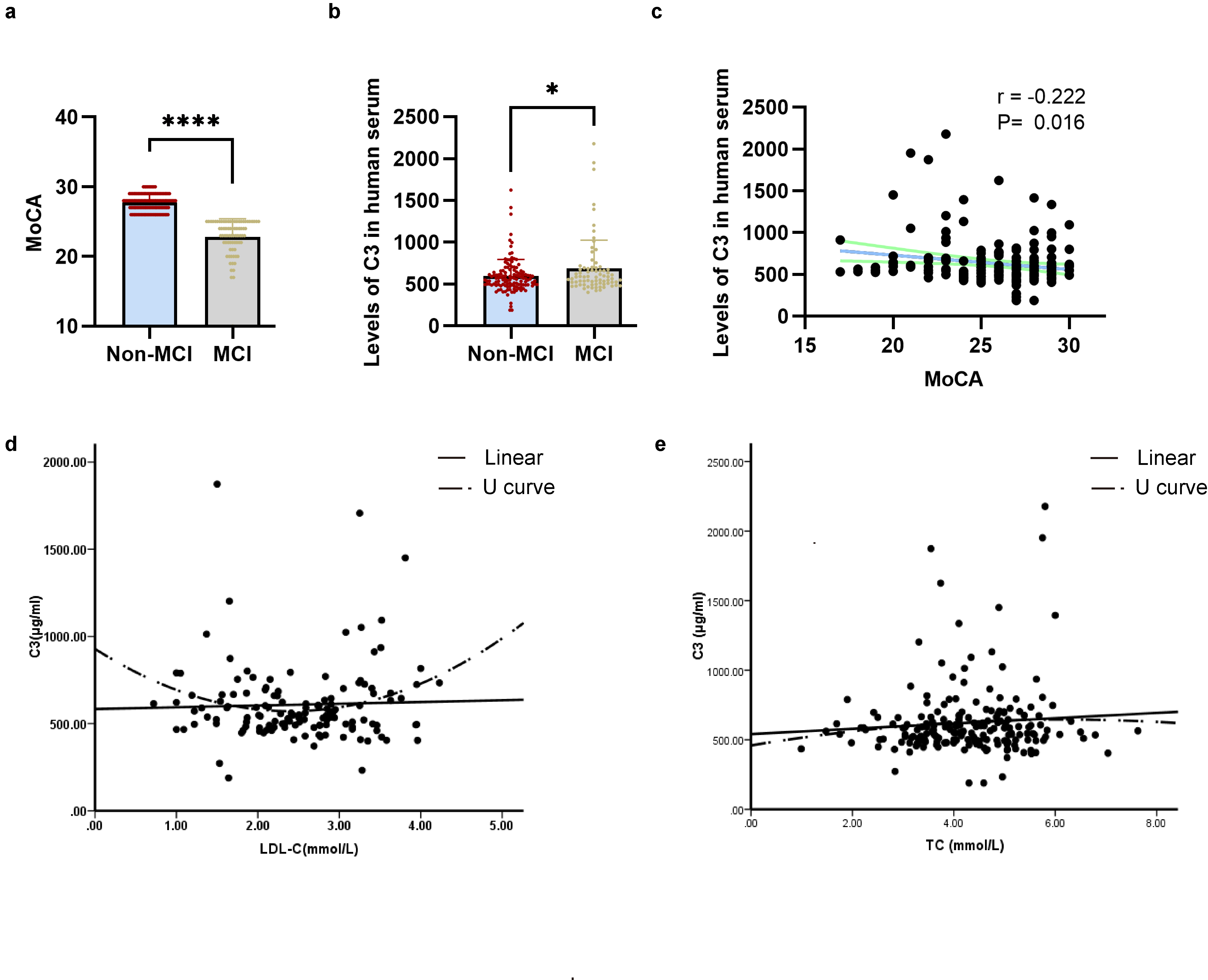
**

**Supplementary Fig. 10**
